# Beyond traditional outbreak investigation: Using genomic data for enhanced detection of COVID-19 disease clusters in Utah

**DOI:** 10.1371/journal.pone.0342637

**Published:** 2026-07-08

**Authors:** Mary Jewell, Abbey Marye, Bree Barbeau, Kelly Oakeson

**Affiliations:** 1 Division of Population Health, Utah Department of Health and Human Services, Salt Lake City, Utah, United States of America; 2 Utah Public Health Laboratory, Utah Department of Health and Human Services, Salt Lake City, Utah, United States of America; Children’s National Hospital, George Washington University, UNITED STATES OF AMERICA

## Abstract

**Background:**

Traditional disease surveillance, such as manual case investigation, was the primary method for identifying disease clusters during the COVID-19 pandemic. However, the pandemic also provides an opportunity to explore how genomic data can be used to improve cluster detection and response. While genomic data can complement traditional methods, guidelines are needed to integrate genomic data into real-time outbreak response.

**Methods:**

Using binomial and multinomial logistic regression, we compared two methods of disease surveillance in Utah: genomic sequencing of COVID-19 cases and manual case investigation. We evaluated whether these two methods reached the same populations geographically and demographically. Next, we performed genomic clustering using SNP distance thresholds and a logit regression model to identify potential transmission clusters. We compared genomic clusters with epi-identified clusters, defined by manual case investigation, using cluster validation metrics (Adjusted Rand Index, VI), and by assessing biological plausibility (monophyly).

**Results:**

The odds of a case being sequenced varied significantly by jurisdiction and race/ethnicity, with patients in several non-White groups being less likely to undergo sequencing. The genomic clustering methods produced clusters that were notably different from epi-identified clusters. Genomic methods, particularly the logit model, resulted in strong clusters based on metrics of cluster validation and biological plausibility. Analysis of specific epi-defined clusters revealed significant discordance with genomic data. Many large clusters were likely composed of multiple distinct genomic introductions, or contained cases that were not genomically linked.

**Conclusions:**

Genomic data provides an advanced level of resolution for defining disease clusters compared to traditional epidemiological data. The disparities in sequencing coverage necessitate demographically and geographically diverse sampling strategies. Furthermore, it is essential to prioritize sequencing cases in a suspected cluster to maximize the impact of genomic surveillance. Integrating genomic data into epidemiologic investigation enables more precise cluster definitions, strengthening outbreak investigation and public health mitigation.

## Introduction

Genomic epidemiology is the process of using pathogen genomic information to understand the distribution and determinants of infectious disease. Genomic data provide an additional level of data resolution, allowing epidemiologists to understand disease transmission at a finer level, which can influence and improve public health response [1,2]. One potential use of genomic data is to help define disease clusters, or cases connected by a shared transmission history [3–12]. In conjunction with traditional epidemiological data, genomic data can help identify transmission patterns or hotspots, which can guide further public health investigation or policy decisions [5,7,13,14].

Despite the potential utility of genomic data, there is a need for more defined guidelines to integrate genomic data into public health practice for ongoing disease response. The COVID-19 pandemic provides a unique opportunity to refine the best practices for using genomic data for outbreak investigation. Over the course of the COVID-19 pandemic, whole genome sequencing (WGS) technology allowed for an unprecedented amount of pathogen genomic information to be generated, and yet, during the pandemic, most cluster detection by public health agencies was done manually, relying on patient interviews and questionnaires. The additional use of genomic data could advance this work by helping to identify clusters with greater speed and accuracy, and providing insight into disease transmission [7,13,14].

The purpose of this report is to explore the use of genomic data in complement to traditional epidemiological data for identifying disease clusters. In order to do this, we compare different methods of disease surveillance in Utah during the pandemic, including genomic sequencing of COVID-19 cases and manual case investigation. We evaluate whether these two methods reached the same populations within Utah, both geographically and demographically, and whether any populations were overlooked by either method. Next, we use genomic clustering algorithms to identify potential transmission clusters and compare clusters identified with genomic data to those identified using traditional epidemiological data alone. We conclude by providing recommendations to optimize the use of genomic data for cluster detection in future outbreak or pandemic scenarios.

## Materials and methods

### Data collection and inclusion

This is a retrospective analysis utilizing data during March 1, 2020–September 30, 2023. We utilized epidemiologic data from the state of Utah, a state in the southwestern United States of about 3 million residents. We extracted epidemiologic data from the UT-NEDSS (EpiTrax) system, Utah’s disease surveillance system, including demographics such as age, sex, race, ethnicity, and jurisdiction of residence, as well as information about investigation status and outbreak status. This anonymized data was accessed on January 15, 2025.

Genomic data of COVID-19 sequences were collected from NCBI GenBank and GISAID and accessed on March 3, 2025. Sequences were included if they were from the state of Utah and had a collection date between March 1, 2020 and September 30, 2023. Sequences were filtered by submitting lab (Utah Public Health Laboratory, UPHL) and quality metrics to ensure usable samples. This resulted in a total of 22,712 sequences (Alpha = 5,175; Delta = 9,875; Omicron = 7,662). Sequence accession numbers are available in supporting information (S1 Appendix).

### Time periods

The sampling time frame was subdivided into three periods to account for differences in the predominant strain of COVID-19 that was circulating within the state and changes in case investigation protocols over the course of the pandemic. These time periods are defined as Alpha (March 2020–June 2021), Delta (June 2021–December 2021), and Omicron (December 2021–September 2023). These time periods were used for stratified analyses to assess how overall patterns may have changed over time.

### Case investigation and sequencing status

Cases were defined as being interviewed if they were either interviewed over the phone by a case investigator, were interviewed on-site at an in-person testing location, or interacted with the online survey instrument developed by the State of Utah for case investigation. Cases who were not contacted by any of these methods were defined as being not interviewed.

Cases were defined as being sequenced if they had a valid WGS collection date listed. Epidemiologic data from each case were matched to genomic data using the UPHL accession number. Not all diagnostic testing modalities were eligible for sequencing. Polymerase chain reaction (PCR) tests were the most commonly used type of test for sequencing COVID-19 samples, but antigen rapid tests were ineligible for sequencing.

Cases were defined as being both interviewed and sequenced if they were contacted by one of the methods listed above AND had a valid WGS collection date listed. Likewise, cases were defined as being neither interviewed nor sequenced if neither of these criteria apply. For the purposes of multinomial logistic regression, these categories (interviewed only, sequenced only, both interviewed and sequenced, or neither interviewed nor sequenced) are mutually exclusive.

### Logistic regression

We characterized the sample population overall and within each time period using descriptive measures, including frequencies, percentages, and means. Subsequently, we used binomial logistic regression to assess the likelihood of a case being sequenced in each local health jurisdiction in Utah and by demographic factors. Lastly, we used multinomial logistic regression to determine the likelihood of sequencing a case compared to reaching them using other methods, such as a manual or virtual case investigation interview. When applicable during regression analyses, we used the following strata as categorical references: Female; White alone, non-Hispanic; and resident of Salt Lake County. We evaluated and confirmed a linear relationship between continuous predictors and the log-odds of the outcome, and assessed multicollinearity among covariates using Variance Inflation Factors (VIF). Missing data affected less than 3% of total records, so we utilized a complete case approach, and cases with missing data were excluded. We constructed four distinct logistic regression models *a priori* to evaluate our research questions, and we report unadjusted p-values to maximize statistical power for this exploratory analysis [15]. All data cleaning, analysis, and visualization was performed using the R programming language (version 4.5.1).

### Clustering methods

We mapped all downloaded genomes to the SARS-CoV-2 reference genome (GenBank Accession Number: NC_045512.2) using snippy. We used snippy to create multiple sequence alignments, which were subsequently used to create SNP distance matrices using snp_dists. We then used MAPLE to create maximum likelihood trees using these alignments.

We performed genomic clustering using multiple methods. First, we defined clusters using only pairwise SNP distance thresholds of 1, 2, or 3 SNPs. This method is a common way of performing genomic clustering, and these specific SNP thresholds are informed by the SARS-CoV-2 mutation rate and similar analyses in literature [3,7,13,14]. These specific parameters are grounded in the baseline substitution rate of SARS-CoV-2, which is estimated to be approximately 10^−3^ substitutions per site per year, although studies have suggested some variability in this mutation rate [16,17]. The emergence of variants of concern seem to be driven by episodic, short-term increases in the mutation rate, possibly occurring in chronic infections of SARS-CoV-2 [17]. However, the intra-lineage substitution rates seemed to remain similar throughout the time periods considered in this study [16–18]. Ashraf et al. found that genome substitution rates rose slightly during 2021, followed by a decrease in 2022 [16]. Thus, we deem it appropriate to apply the same SNP thresholds of 1, 2, and 3 SNPs across all three time periods, allowing for standardized comparisons of cluster topology across time periods. We used the hclust function from the cluster package (version 2.1.8.1) to perform hierarchical clustering at these defined thresholds in R.

Next, we used a logit regression model that clusters cases using a pairwise probability threshold. This method incorporates both genomic relatedness as well as temporal distance through sample collection date, and it can allow for the inclusion of other variables of interest, potentially resulting in more meaningful clusters. We applied this method using the publicly available R package cov2clusters, taking into account genomic distance and temporal distance. We used probability threshold parameters of 0.8 and 0.9, as these have been shown to have high precision and recall [6]. These parameters allow for consistently useful results throughout the three time periods we consider. During high incidence periods like Omicron, there may be more identical lineages circulating independently in the population, so requiring both a high genetic and temporal probability reduces the risk of falsely aggregating unrelated clusters.

In comparison, epidemiologically defined clusters were defined as a group of cases belonging to the same outbreak in Utah’s disease surveillance system. These outbreaks were made by public health investigators during the case investigation process, taking into account factors such as common exposures, travel, and workplace or school affiliation. These data were collected through interviews conducted by investigators or through patient interaction with the online survey instrument.

### Cluster validation analysis

For this investigation, there are no “gold standard” clustering labels, as it is ultimately unknown which “true” cluster a case may belong to. Consequently, rather than assessing external validation of clusters, or the comparison of clustering methods to pre-existing labels, we assessed internal validation, which is a measure of the compactness and separation of clusters. As a metric for internal validation, we computed the mean silhouette score of each genomic clustering method in each time period (Alpha, Delta, or Omicron). A silhouette score measures how similar each sample is to its own cluster compared to other clusters, and the mean scores range from −1 (very weak clustering) to 1 (very strong clustering) [19].

In addition to internal validation, we also assessed relative validation, or the extent to which each clustering method differed from the others. This provides insight into the extent to which different clustering methods agree with each other. In order to assess the degree of agreement between different clustering methods, we computed pairwise adjusted Rand index (ARI) and variation of information (VI) metrics between each clustering method, both epidemiologically defined and genomic methods. Both ARI and VI assess the similarity between clusterings, but they capture different aspects of similarity. ARI is based on pairwise comparison and assesses whether there is agreement in pairwise sample co-membership [20]. On the other hand, VI is a distance metric that quantifies entropy and how much information is lost or gained when moving from one clustering to another [21]. Together, these metrics provide insight into the similarity or dissimilarity of different clustering methods.

Lastly, we investigated the biological plausibility of each identified cluster by assessing whether genomic and epidemiologically identified clusters were monophyletic. Monophyletic groups include all descendants from a single most recent common ancestor, indicating a close genomic relationship—and thus a likely epidemiologic relationship— between cases in the group. We used the ape package (version 5.8−1) in R to assess whether each identified cluster is monophyletic according to the maximum likelihood trees we developed.

### Investigating epidemiologic clusters

Finally, we selected several epidemiologically defined clusters to assess whether there were genomic links supporting their clustering. Clusters were selected based on the number of sequenced samples they included, thus excluding very small clusters and very large clusters that had a small proportion of sequenced samples. Clusters were selected for evaluation if they included 30 or more sequenced samples. We calculated the mean SNP difference within each cluster and visualized each cluster as a phylogenetic tree annotated with the samples’ genomic clustering results.

### Ethical approval

As this study used publicly available pathogen genomic data and anonymized patient demographic data, formal ethical approval was not necessary.

## Results

### Sample characterization

A total of 697,393 cases from Utah’s disease surveillance system were included in this analysis. Of these, 391,054 cases (56.1%) were contacted for an interview but not sequenced, while 34,882 cases (5.0%) were sequenced without being contacted for an interview. An additional 41,983 cases (6.0%) were both interviewed and sequenced, while 229,474 cases (32.9%) were neither contacted for an interview nor sequenced.

The average age of cases was 35.1, and the majority of the cases included in this analysis were female (n = 356,250; 51.1%) and identified as White, non-Hispanic (n = 500,596; 71.8%). The most common jurisdiction of residence for cases included in this analysis was Salt Lake County (192,503; 27.6%), followed by Utah County (n = 169,357; 24.3%), the two most populous counties. These characteristics are summarized in Table 1.

**Table 1:** Demographic characteristics of cases included stratified by time period.

	Overall	Alpha	Delta	Omicron
**Sex**				
Male	340,632 (48.8%)	191,157 (49.6%)	88,320 (48.8%)	60,574 (46.7%)
Female	356,250 (51.1%)	194,193 (50.4%)	92,480 (51.1%)	68,909 (53.1%)
Unknown/missing	511 (0.07%)	109 (0.03%)	148 (0.08%)	253 (0.2%)
**Race and ethnicity**				
American Indian/Alaska Native alone, non-Hispanic	9,284 (1.3%)	4,231 (1.09%)	2,073 (1.1%)	2,540 (2.0%)
Asian alone, non-Hispanic	13,420 (1.9%)	7,514 (1.9%)	3,132 (1.7%)	2,768 (2.1%)
Black/African American alone, non-Hispanic	8,464 (1.2%)	4,808 (1.2%)	2,044 (1.1%)	1,598 (1.2%)
Native Hawaiian/Pacific Islander alone, non-Hispanic	12,949 (1.9%)	9,002 (2.3%)	2,649 (1.5%)	1,293 (1.0%)
White alone, non-Hispanic	500,596 (71.8%)	264,033 (68.5%)	138,990 (76.8%)	96,948 (74.7%)
Hispanic or Latino (any race)	125,726 (18%)	81,863 (21.2%)	24,423 (13.5%)	19,315 (15.0%)
Two or more races, non-Hispanic	1,282 (0.8%)	950 (0.2%)	209 (0.1%)	123 (0.1%)
Some other race alone, non-Hispanic	11,333 (1.6%)	6,526 (1.7%)	2,713 (1.5%)	2,076 (1.6%)
Unknown race, non-Hispanic	14,339 (2.1%)	6,532 (1.7%)	4,715 (2.6%)	3,075 (2.4%)
**Age**				
Mean (SD)	35.1 (20.0)	35.3 (18.6)	32.7 (20.9)	37.9 (22.3)
**Jurisdiction**				
Bear River	43,464 (6.2%)	20,435 (5.3%)	11,962 (6.6%)	11,044 (8.5%)
Central Utah	15,680 (2.2%)	8,195 (2.1%)	6,194 (3.4%)	1,276 (1.0%)
Davis County	75,205 (10.8%)	38,420 (10.0%)	22,584 (12.5%)	14,145 (10.9%)
San Juan	4,402 (0.6%)	1,524 (0.4%)	779 (0.4%)	1,658 (1.3%)
Salt Lake County	192,503 (27.6%)	148,556 (38.5%)	39,413 (21.8%)	4,422 (3.4%)
Southeast Utah	9,315 (1.3%)	3,457 (0.9%)	2,929 (1.6%)	2,921 (2.2%)
Southwest Utah	52,072 (7.5%)	26,809 (7.0%)	14,611 (8.0%)	10,528 (8.1%)
Summit County	9,635 (1.4%)	4,676 (1.2%)	2,691 (1.5%)	2,261 (1.7%)
Tooele County	14,796 (2.1%)	6,261 (1.6%)	5,537 (3.1%)	2,986 (2.3%)
TriCounty	8,761 (1.3%)	3,623 (0.9%)	4,121 (2.3%)	999 (0.8%)
Utah County	169,357 (24.3%)	87,494 (22.7%)	45,835 (25.3%)	35,936 (27.7%)
Wasatch County	8,426 (1.2%)	4,512 (1.2%)	2,304 (1.3%)	1,597 (1.2%)
Weber-Morgan	90,558 (13.0%)	30,933 (8.0%)	21,256 (11.7%)	38,042 (29.3%)
**How was the case reached?**				
Interviewed only	391,054 (56.1%)	278,487 (72.2%)	78,536 (43.4%)	33,539 (25.9%)
Sequenced only	34,882 (5.0%)	2,983 (0.8%)	16,622 (9.2%)	15,133 (11.7%)
Both interviewed and sequenced	41,983 (6.0%)	9,763 (2.5%)	21,642 (12.0%)	10,485 (8.1%)
Neither	229,474 (32.9%)	94,226 (24.4%)	64,148 (35.5%)	70,579 (54.4%)

**Fig 1:**
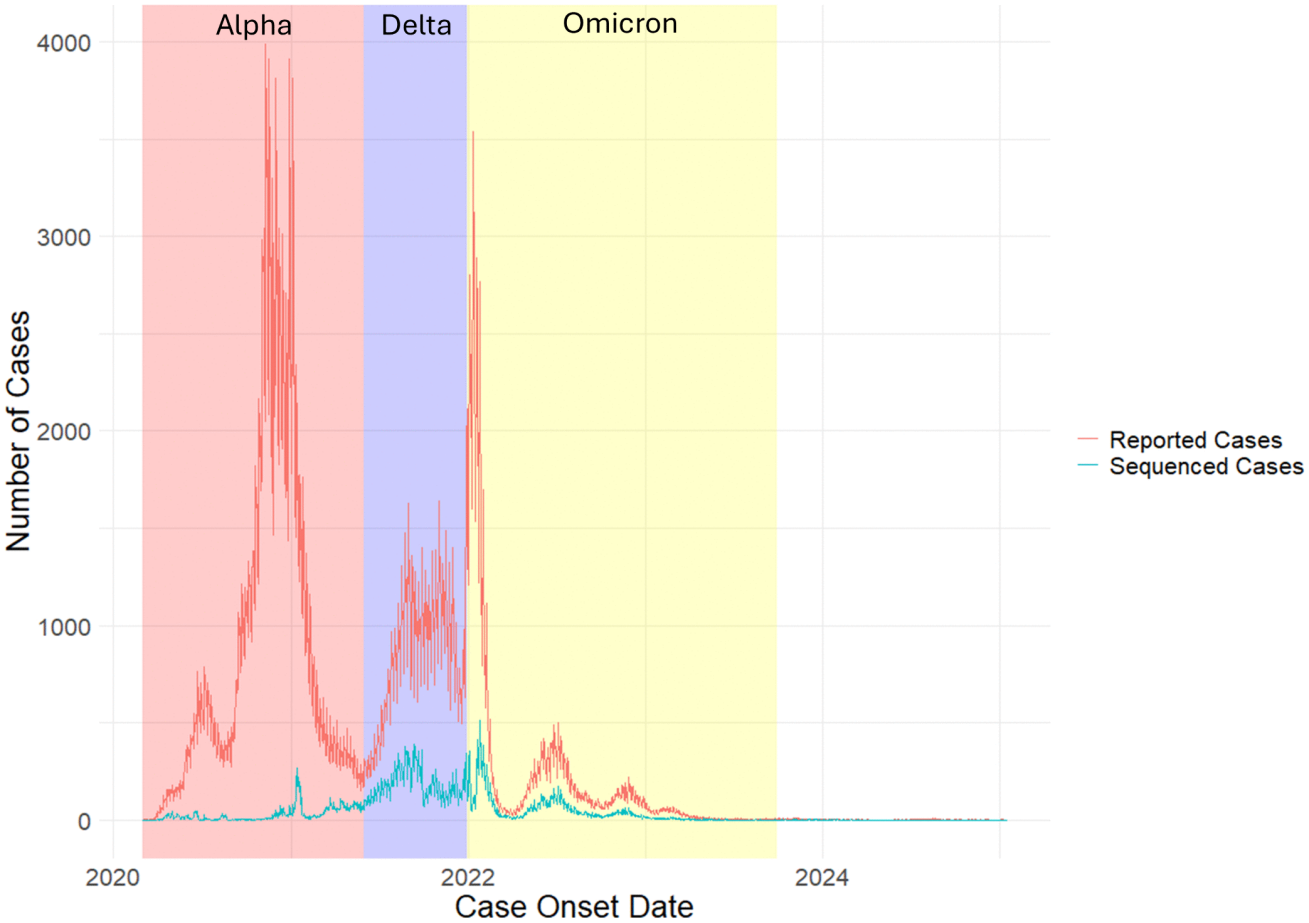
The number of reported cases (red) and sequenced cases (blue) recorded over the entire study period, including the Alpha, Delta, and Omicron periods. The Alpha period (March 2020–June 2021) is shaded pink, the Delta period (June 2021–December 2021) is shaded purple, and the Omicron period (December 2021–September 2023) is shaded yellow.

The proportion of reported cases for which there was sequencing data varied throughout the study period (Fig 1). On average, cases during the Delta time period (during June 2021–December 2021) were most likely to be sequenced.

### Surveillance methods by demographics and jurisdiction

**Fig 2:**
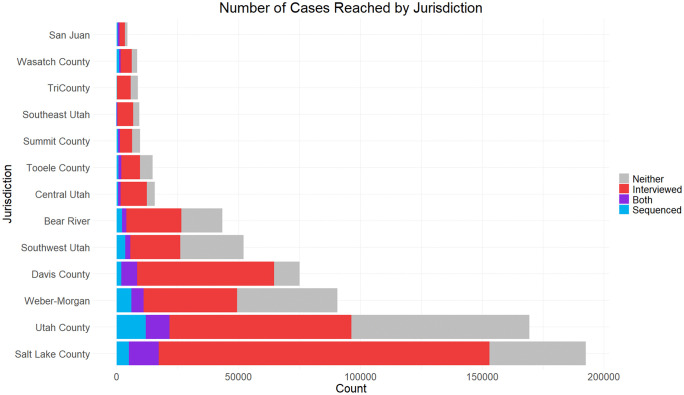
The number of cases interviewed (red), sequenced (blue), both interviewed and sequenced (purple), or neither (gray) in each jurisdiction.

The number and proportion of cases being interviewed, sequenced, or both differs by jurisdiction of residence. Salt Lake County had the greatest number of cases overall (n = 192,503; 27.6% of total) and the greatest number of cases that were interviewed (Fig 2). However, Salt Lake County did not have the greatest number of sequenced cases among all jurisdictions (n = 17,244), and it had among the lowest proportion of cases sequenced (9%). Conversely, several rural jurisdictions with low overall case numbers had the highest proportion of sequenced cases, including San Juan (28% of cases sequenced), followed by Wasatch County (20% of cases sequenced).

**Fig 3:**
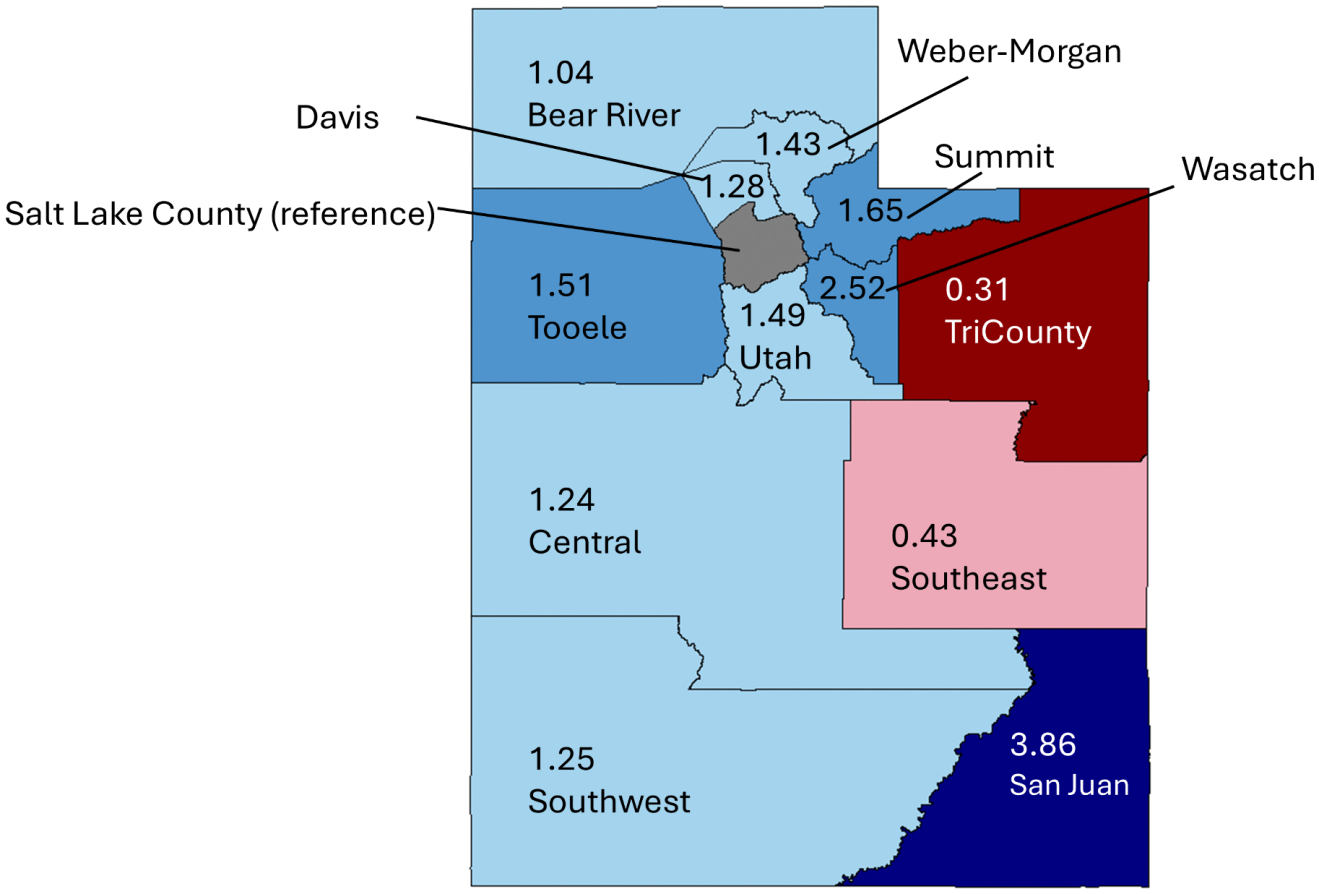
Odds ratios (ORs) of sequencing a case in each health jurisdiction throughout Utah, using Salt Lake County as a reference. Jurisdictions with ORs > 1 are represented in blue, while jurisdictions with ORs < 1 are represented in red. The base map shapefile was obtained from Utah Automated Geographic Reference Center (AGRC) and is used under a Creative Commons Attribution 4.0 International License (CC BY 4.0), original copyright November 21, 2019. Referent category: Salt Lake County. * p-value ≤ 0.05. ** p-value ≤ 0.01. *** p-value ≤ 0.001.

Binomial logistic regression shows that there are differences in the odds of a case being sequenced according to patient racial and ethnic identity and jurisdiction of residence. The odds of a case being sequenced, as opposed to not being sequenced, are higher in most health jurisdictions throughout the state compared to cases in Salt Lake County (Fig 3). The exceptions are Southeast Utah and TriCounty, where the odds of a case being sequenced are 57% and 69% lower respectively compared to Salt Lake County. The jurisdiction with the highest odds of a case being sequenced is San Juan, a jurisdiction with a high American Indian/Alaska Native population, with cases there having 286% higher odds of being sequenced compared to cases in Salt Lake County.

**Fig 4:**
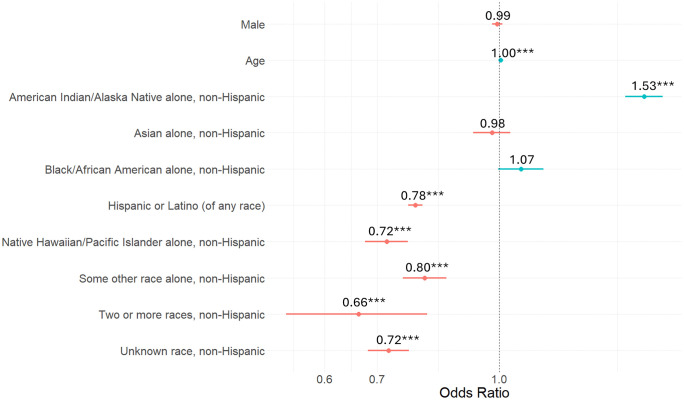
Adjusted odds of sequencing a case across the sampling period. Referent categories: Female; White alone, non-Hispanic. * p-value ≤ 0.05. ** p-value ≤ 0.01. *** p-value ≤ 0.001.

The odds of a case being sequenced, as opposed to not sequenced, are also lower among several non-White racial and ethnic identities compared to those of the White, non-Hispanic group (Fig 4). However, the odds of a case being sequenced among those identifying as American Indian/Alaska Native are 53% higher than among those identifying as non-Hispanic White.

**Fig 5:**
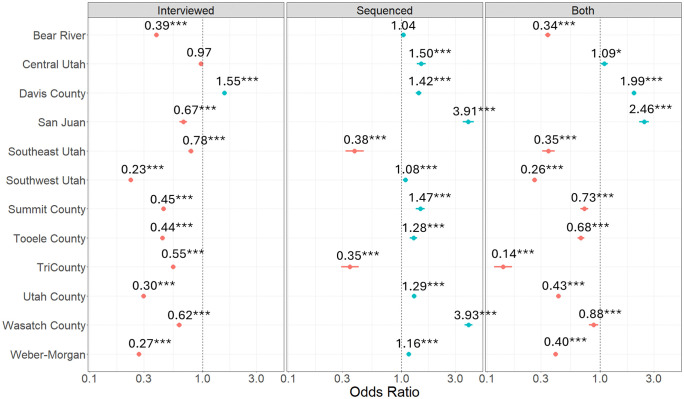
Odds of a case successfully undergoing sequencing only, interview only, or both, as compared to neither interview nor sequencing, across the sampling period. Referent category: Salt Lake County. * p-value ≤ 0.05. ** p-value ≤ 0.01. *** p-value ≤ 0.001.

Multinomial logistic regression also shows differences in the odds of a case being interviewed, sequenced, or both by jurisdiction and racial and ethnic identity, as opposed to being neither interviewed nor sequenced. Cases in most jurisdictions were less likely to be interviewed only compared to those in Salt Lake County, but conversely, with the exception of Southeast and TriCounty, cases in most other jurisdictions were more likely to be sequenced only compared to those in Salt Lake County (Fig 5). Three jurisdictions were more likely to be both sequenced and interviewed compared to Salt Lake County, including Central (9% higher odds), Davis County (99% higher odds), and San Juan (146% higher odds) (Fig 5).

**Fig 6:**
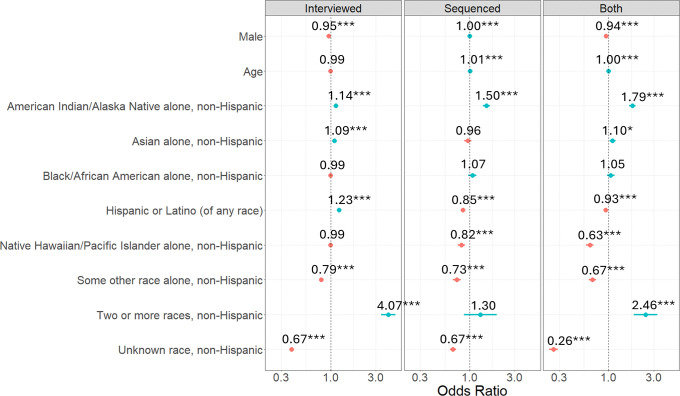
Adjusted odds of a case successfully undergoing sequencing only, interview only, or both, as compared to neither interview nor sequencing, across the sampling period. Referent categories: Female; White alone, non-Hispanic. * p-value ≤ 0.05. ** p-value ≤ 0.01. *** p-value ≤ 0.001.

There were also differences in surveillance methods by racial and ethnic identity, with several racial groups being more likely to be both interviewed and sequenced compared to those identifying as White, non-Hispanic (Fig 6). Specifically, cases identifying as American Indian/Alaska Native, Asian, Black/African American, or two or more races had greater odds of being both interviewed and sequenced rather than being neither interviewed nor sequenced. There were generally no significant differences in the odds of being both interviewed and sequenced by age or sex, although compared to females, males were slightly less likely to be interviewed (5% lower odds) or both interviewed and sequenced (6% lower odds).

### Case clustering

#### Epidemiologic case clustering.

There were 8,299 unique epidemiologically identified clusters named in Utah’s disease surveillance system, including 3,476 clusters in workplace settings and 1,953 clusters in school settings. The largest epidemiologically identified cluster was associated with a correctional facility, and included 1,707 cases. The mean number of cases per cluster was 84, while the median number of cases per cluster was three.

Of the 8,299 epidemiologically identified clusters, only 222 (2.7%) had all cases within the cluster sequenced. There were 1,012 clusters where 50% or more of the cases were sequenced, representing 12.2% of the epidemiologically identified clusters. The average epidemiologically identified cluster had sequencing data for 10.9% of its cases.

#### SNP threshold clustering.

**Fig 7:**
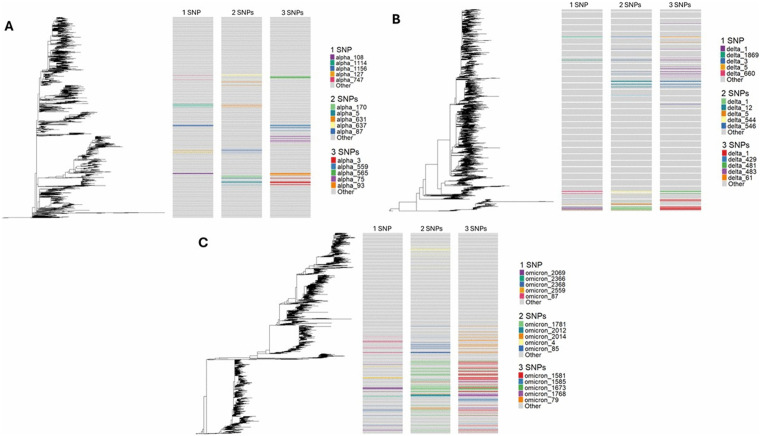
Maximum likelihood phylogenetic trees illustrating the genomic diversity of SARS-CoV-2 samples during the Alpha time period (March 2020–June 2021) (A), Delta time period (June 2021–December 2021) (B), and Omicron time period (December 2021–September 2023) (C). Columns of bars next to each tree represent the cluster assignment for each tree tip using SNP distance clustering and SNP thresholds of 1, 2, and 3 SNPs. Colored bars represent the five largest clusters identified by each method, while gray bars indicate samples that belong to smaller clusters, and white bars represent samples that did not belong to any particular cluster.

In general, SNP threshold clustering identified more clusters than were identified using epidemiologic methods alone. Using a 1 SNP threshold identified 14,536 clusters, while a 2 SNP threshold yielded 11,489 clusters, and a 3 SNP threshold resulted in 9,736 clusters. However, this method places every sample into a cluster, even if that cluster only consists of one or two samples; this method results in more identified clusters by design. The average cluster size with a 1 SNP threshold was 1.6 cases, while the average cluster size with a 2 SNP and 3 SNP threshold was 2.0 and 2.3 cases respectively (Fig 7).

#### Logit model clustering.

**Fig 8:**
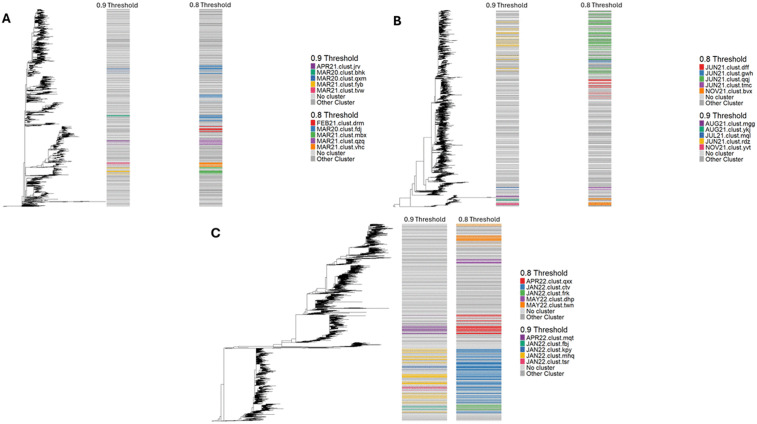
Maximum likelihood phylogenetic trees illustrating the genomic diversity of SARS-CoV-2 samples during the Alpha time period (March 2020–June 2021) (A), Delta time period (June 2021–December 2021) (B), and Omicron time period (December 2021–September 2023) (C). Columns of bars next to each tree represent the cluster assignment for each tree tip using the cov2clusters logit model and a probability threshold of 0.9 or 0.8. Colored bars represent the five largest clusters identified by each method, while gray bars indicate samples that belong to smaller clusters, and white bars represent samples that did not belong to any particular cluster.

Clustering using the cov2clusters logit model resulted in fewer clusters identified than either SNP threshold clustering or epidemiologically identified clusters. Since this method incorporates both temporal distance and genomic distance, it is likely to identify fewer clusters by design, though the clusters it identifies may be more accurate because of the added information. Using a probability threshold of 0.8, the model identified 2,215 unique clusters with an average of 10.3 cases per cluster, while a probability threshold of 0.9 resulted in 2,073 clusters with an average of 11.0 cases per cluster (Fig 8).

### Cluster validation metrics and analyses

As a measure of internal validity, we computed the average silhouette score for each genomic clustering method, including SNP threshold clustering with a threshold of 1, 2, or 3 SNPs (SNP 1, SNP 2, SNP 3), and logit model clustering using cov2clusters and probability thresholds of 0.8 or 0.9 (cov08 and cov09). These mean silhouette scores were computed for clusters within each time period (Alpha, Delta, or Omicron) (Table 2).

**Table 2:** Mean silhouette scores for each genomic clustering method, including SNP threshold clustering (SNP 1, SNP 2, SNP 3), and logit model clustering using cov2clusters (cov08 and cov09) in each time period (Alpha, Delta, or Omicron).

Time period	Clustering method	Mean silhouette score
**Alpha**	SNP 1	0.34
	SNP 2	0.28
	SNP 3	0.27
	cov08	0.14
	cov09	0.34
**Delta**	SNP 1	0.28
	SNP 2	0.22
	SNP 3	0.21
	cov08	0.17
	cov09	0.39
**Omicron**	SNP 1	0.21
	SNP 2	0.14
	SNP 3	0.12
	cov08	0.44
	cov09	0.56

The highest mean silhouette score within each time period was from the logit method using a probability threshold of 0.9 (cov09). SNP threshold methods generally had the next-highest mean silhouette scores, with a 1-SNP threshold generally resulting in higher mean silhouette scores than a threshold of 2 or 3 SNPs. In general, many of the mean silhouette scores were in the range of 0.25–0.50, indicating weak to moderate clustering, although silhouette scores are known to perform poorly if data clusters have irregular shapes and sizes.

We also computed pairwise adjusted Rand index (ARI) metrics between each method considered here, including epidemiological clustering (Epi), SNP threshold clustering (SNP 1, SNP 2, and SNP 3), and logit model clustering using cov2clusters (cov08 and cov09) (Table 3). The highest ARI values are between the SNP clusterings (SNP 1, SNP 2, and SNP 3), and, separately, between the logit model clusterings (cov08 and cov09). By contrast, the ARI values comparing the SNP clusterings with the logit model clusterings were all near zero, indicating very poor agreement between these different methods. Similarly, pairwise comparisons show that the epidemiological grouping has generally poor agreement with all of the genomic methods, but slightly better agreement with the SNP threshold clusterings than the logit model clusterings.

**Table 3:** Pairwise adjusted Rand index (ARI) metrics between each method, including epidemiological clustering (Epi), SNP threshold clustering (SNP 1, SNP 2, SNP 3), and logit model clustering using cov2clusters (cov08 and cov09).

	Epi	SNP 1	SNP 2	SNP 3	cov08	cov09
Epi		0.152	0.190	0.194	0.0111	0.000647
SNP 1	0.152		0.310	0.237	0.000872	0.0000800
SNP 2	0.190	0.310		0.609	0.00380	−0.000137
SNP 3	0.194	0.237	0.609		0.00392	−0.000372
cov08	0.0111	0.000872	0.00380	0.00392		0.462
cov09	0.000647	0.0000800	−0.000137	−0.000372	0.462	

Additionally, we computed pairwise variation of information (VI) metrics between each clustering method and found that the lowest VIs were between the 1-, 2-, and 3-SNP threshold clustering methods, indicating that these clusterings are the most similar to each other (Table 4). The largest differences occur between the logit regression models and the SNP threshold methods. All genomic clustering methods yielded groupings that differed from epidemiologically identified clusters, with pairwise VI values ranging from 1.12 (1-SNP threshold vs epi-identified clusters) to 4.31 (logit regression model vs epi-identified clusters).

**Table 4:** Pairwise variation of information (VI) metrics between each method, including epidemiological clustering (Epi), SNP threshold clustering (SNP 1, SNP 2, SNP 3), and logit model clustering using cov2clusters (cov08 and cov09).

	Epi	SNP 1	SNP 2	SNP 3	cov08	cov09
Epi		1.12	1.23	1.29	3.53	4.31
SNP 1	1.12		0.806	1.08	5.43	6.82
SNP 2	1.23	0.806		0.682	5.20	6.67
SNP 3	1.29	1.08	0.682		5.17	6.59
cov08	3.53	5.43	5.20	5.17		2.38
cov09	4.31	6.82	6.67	6.59	2.38	

In general, compared to epidemiologically identified clusters, genomic methods resulted in low ARI values (range: 0.00065–0.46) and high VI values (range: 1.12–4.31), indicating pronounced differences in clustering structure between genomic and epidemiologic clusterings.

#### Biological plausibility of clusters.

We investigated the biological plausibility of clusters by assessing whether each cluster was monophyletic, meaning it includes all descendants from a common ancestor. We found that very few epidemiologically identified clusters were monophyletic, which suggests that many epidemiologic clusters were either incomplete, missing cases that were potentially linked, or too broad, including cases that were likely not genomically related. In comparison, both SNP-threshold and logit model genomic clustering methods resulted in clusters that were much more likely to be monophyletic (Table 5). In particular, the logit model methods had a much higher proportion of monophyletic clusters identified compared to epidemiologic methods alone.

**Table 5:** The number and proportion of monophyletic clusters identified by each method, including epidemiological clustering (Epi), SNP threshold clustering (SNP 1, SNP 2, SNP 3), and logit model clustering using cov2clusters (cov08 and cov09). Proportions are calculated as the number of monophyletic clusters identified out of the total number of clusters identified by that method in that time period.

	Number of monophyletic clusters (%)			
Clustering method	Alpha	Delta	Omicron	Overall
Epi	20 (0.4%)	16 (0.6%)	2 (0.2%)	38 (0.5%)
SNP1	485 (15.4%)	781 (12.8%)	607 (11.4%)	1873 (12.9%)
SNP2	436 (17.3%)	733 (15.5%)	576 (13.5%)	1745 (15.1%)
SNP3	410 (19.1%)	699 (17.8%)	516 (14.0%)	1625 (16.7%)
cov08	311 (56.5%)	612 (57.7%)	423 (68.3%)	1346 (60.8%)
cov09	267 (53.9%)	561 (58.2%)	381 (61.6%)	1209 (58.3%)

It is possible that the improved recognition of monophyletic clusters for genomic methods is due in part to the fact that genomic clusters by definition include only cases that were sequenced, whereas not all cases in epidemiological clusters may have been sequenced. However, this does provide an indicator that the genomically-identified clusters are generally biologically plausible clusters of linked cases.

### Identifying genomic links in epidemiologically identified clusters

We selected 13 epidemiologically defined clusters to evaluate whether genomic evidence supports their clustering. Though we selected clusters with a minimum of 30 sequenced samples, not all sequences were available for analysis; the clusters we were able to analyze included a minimum of 10 and maximum of 93 sequenced samples. The mean SNP differences of these clusters ranged from 1.60 to 41.63 SNPs.

**Fig 9:**
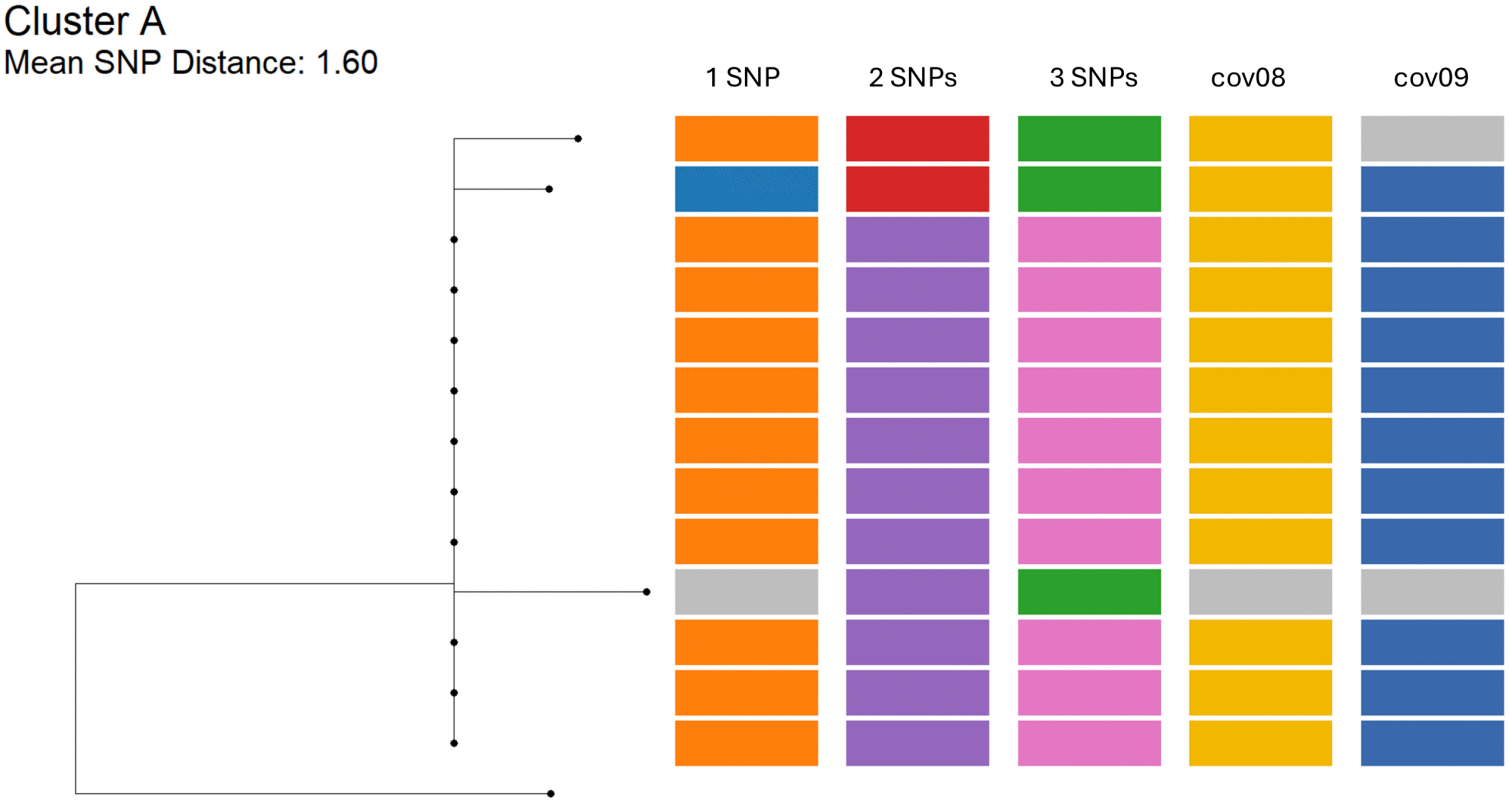
A maximum likelihood phylogenetic tree representing Cluster A, an epidemiologically defined outbreak associated with a nursing home. Cluster A includes 60 cases, of which 13 sequences are represented in this tree. The colors in each column represent cluster membership as identified by each method, including SNP threshold clustering (SNP 1, SNP 2, SNP 3), and logit model clustering using cov2clusters (cov08 and cov09). Gray bars represent sequences that were not part of any cluster according to that method.

In general, our analysis showed discordance between the groups defined by epidemiologic investigation and those defined by genomic linkages. However, in some smaller clusters, there was some agreement between both the epidemiological clustering and all methods of genomic clustering. One of the best examples of this is “Cluster A,” a cluster from April 2020 that was associated with a nursing home (Fig 9).

The epidemiologically defined outbreak for “Cluster A” included 60 cases, but due to the availability of sequences, 13 samples are included in this phylogenetic tree. This cluster has the smallest mean SNP distance of all the clusters in this analysis, and it is one of the best examples of concordance between epidemiologically defined clusters and genomic clustering methods. With the potential exception of one or two samples, all genomic clustering methods seem to support the grouping of these cases.

However, not all clusters were as well defined. There were several large clusters that took place in correctional facility settings, which serve to illustrate the different insights that genomic data can provide to epidemiologic outbreak investigation.

**Fig 10:**
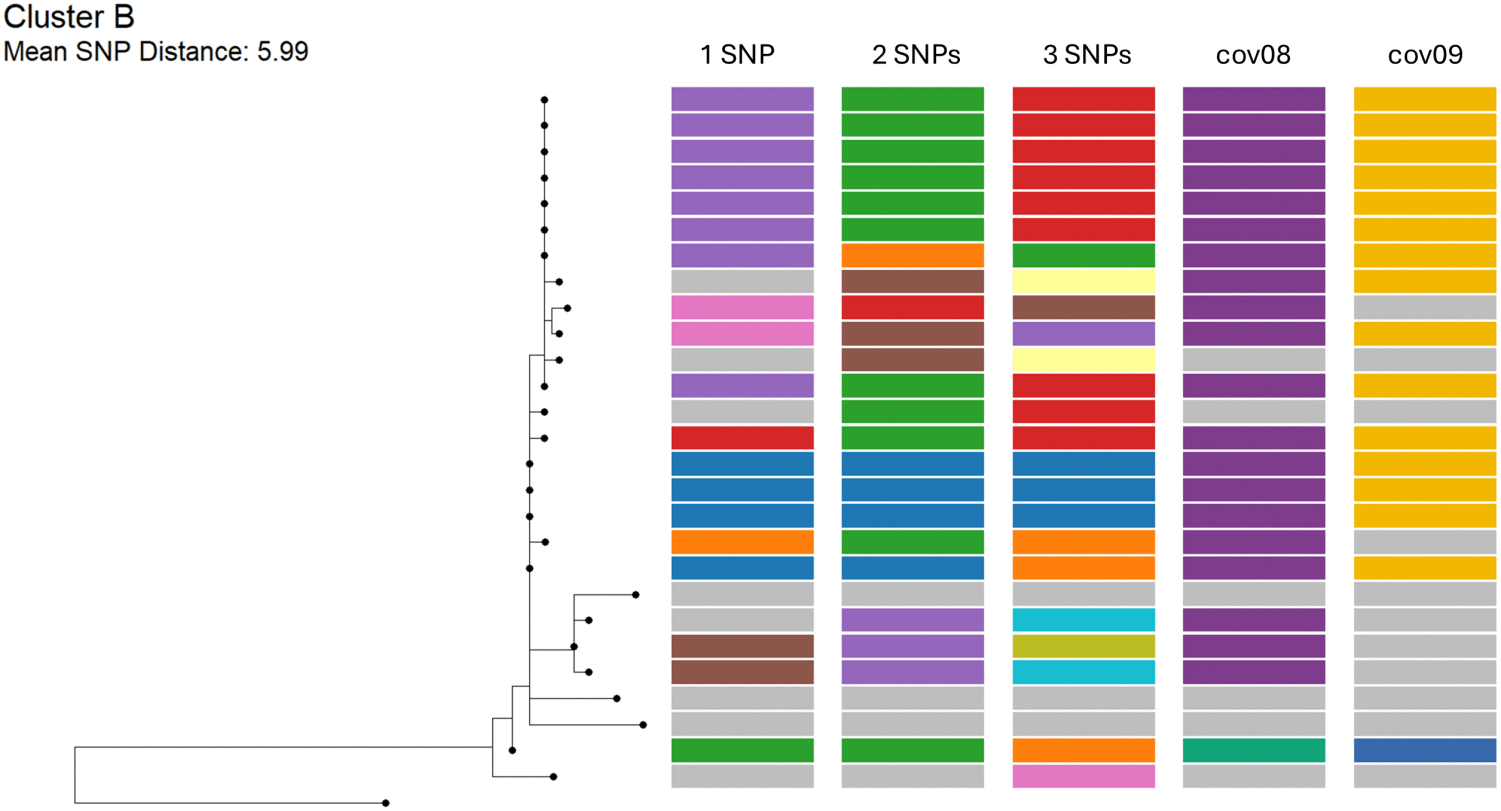
A maximum likelihood phylogenetic tree representing Cluster B, an epidemiologically defined outbreak associated with a correctional facility. Cluster B includes 48 cases, of which 27 sequences are represented in this tree. The colors in each column represent cluster membership as identified by each method, including SNP threshold clustering (SNP 1, SNP 2, SNP 3), and logit model clustering using cov2clusters (cov08 and cov09). Gray bars represent sequences that were not part of any cluster according to that method.

For example, “Cluster B” took place in a state prison setting in August 2021 (Fig 10). The full cluster as it was defined epidemiologically included 48 cases, but 27 samples are represented on the tree. Though there is not a consensus between the different genomic clustering methods, all methods seem to show that there is at least one genomic cluster represented here. However, it also seems to show there are several samples included on this tree that are not actually connected to this cluster genomically. The addition of these genomic data during contact tracing or case investigation could have potentially refined the epidemiologic definition of this cluster.

**Fig 11:**
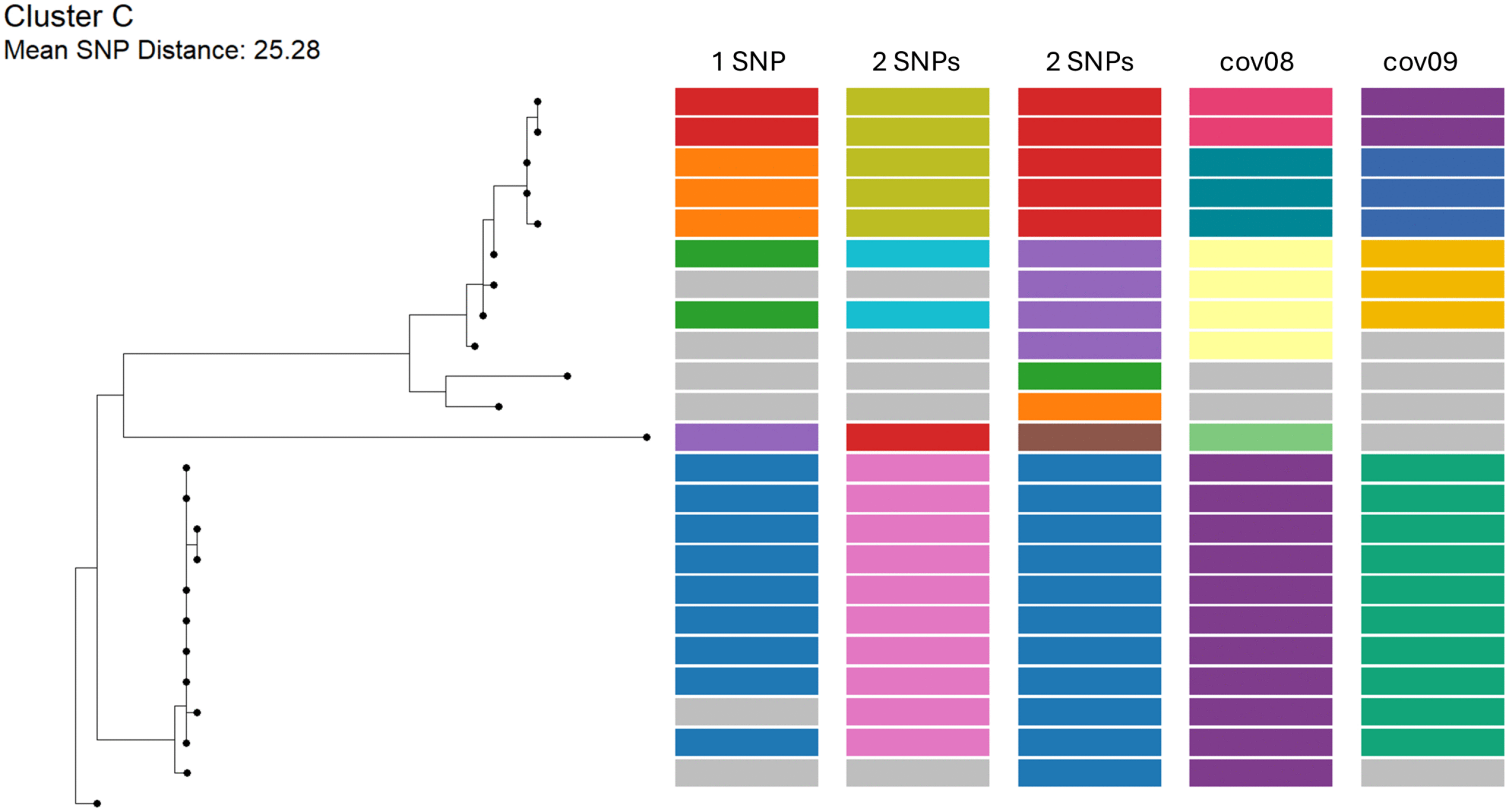
A maximum likelihood phylogenetic tree representing Cluster C, an epidemiologically defined outbreak associated with a correctional facility. Cluster C includes 256 cases, of which 23 sequences are represented in this tree. The colors in each column represent cluster membership as identified by each method, including SNP threshold clustering (SNP 1, SNP 2, SNP 3), and logit model clustering using cov2clusters (cov08 and cov09). Gray bars represent sequences that were not part of any cluster according to that method.

Similarly, “Cluster C” took place in a county jail in June 2020, and it included 256 cases, although this phylogenetic tree represents 23 sequences (Fig 11). Comparing each method of genomic clustering with these cases, the exact cluster membership differs slightly between methods, but all methods identify two or more distinct genomic clusters in this group. It seems likely that there were multiple introductions into the jail and then onward spread from there, but due to a lack of data resolution when defining this outbreak, all cases in the jail were grouped together.

**Fig 12:**
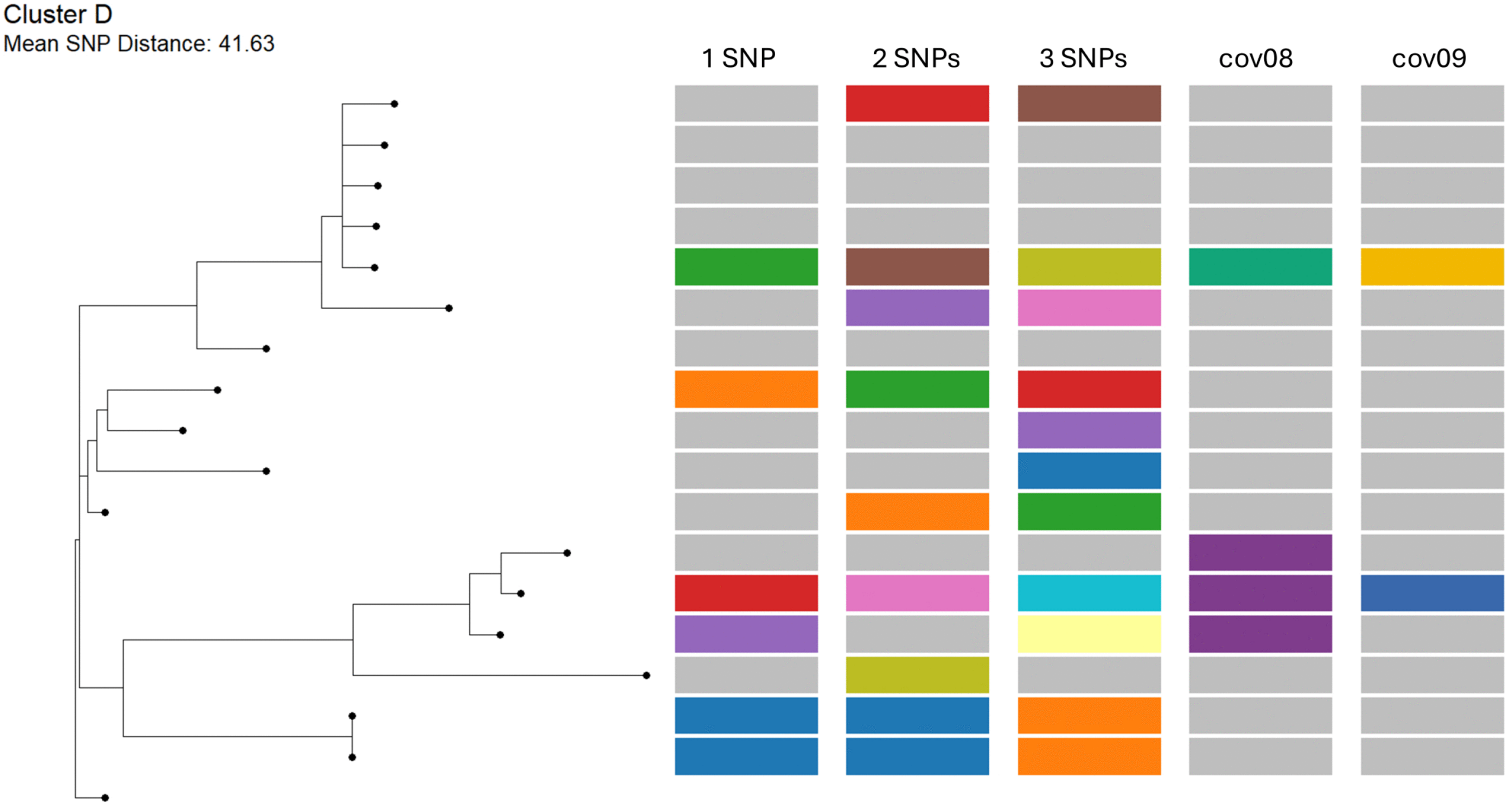
A maximum likelihood phylogenetic tree representing Cluster D, an epidemiologically defined outbreak associated with a correctional facility. Cluster D includes 288 cases, of which 17 sequences are represented in this tree. The colors in each column represent cluster membership as identified by each method, including SNP threshold clustering (SNP 1, SNP 2, SNP 3), and logit model clustering using cov2clusters (cov08 and cov09). Gray bars represent sequences that were not part of any cluster according to that method.

In comparison, “Cluster D,” which took place in April 2020 in a county jail setting, showed very little evidence of a genomic link between cases (Fig 12). As it was defined epidemiologically, this cluster contained 288 cases, though 17 are represented on this tree.

Cluster D had the largest mean SNP difference of all the clusters we analyzed here (41.63 SNPs), and each of the five genomic clustering methods fails to identify clear links between the cases. However, several of the cases on this tree were identified as belonging to a genomic cluster whose other members are not represented on this tree. Therefore, it seems likely that this “cluster” was composed of cases connected to other groups in the community before they entered the jail, and in fact there are very few connections between individuals within the jail. However, it is also worth emphasizing that this tree only represents a small fraction of the total cases in this cluster as it was defined epidemiologically; if more sequences were available for analysis, the data may tell a different story.

**Fig 13:**
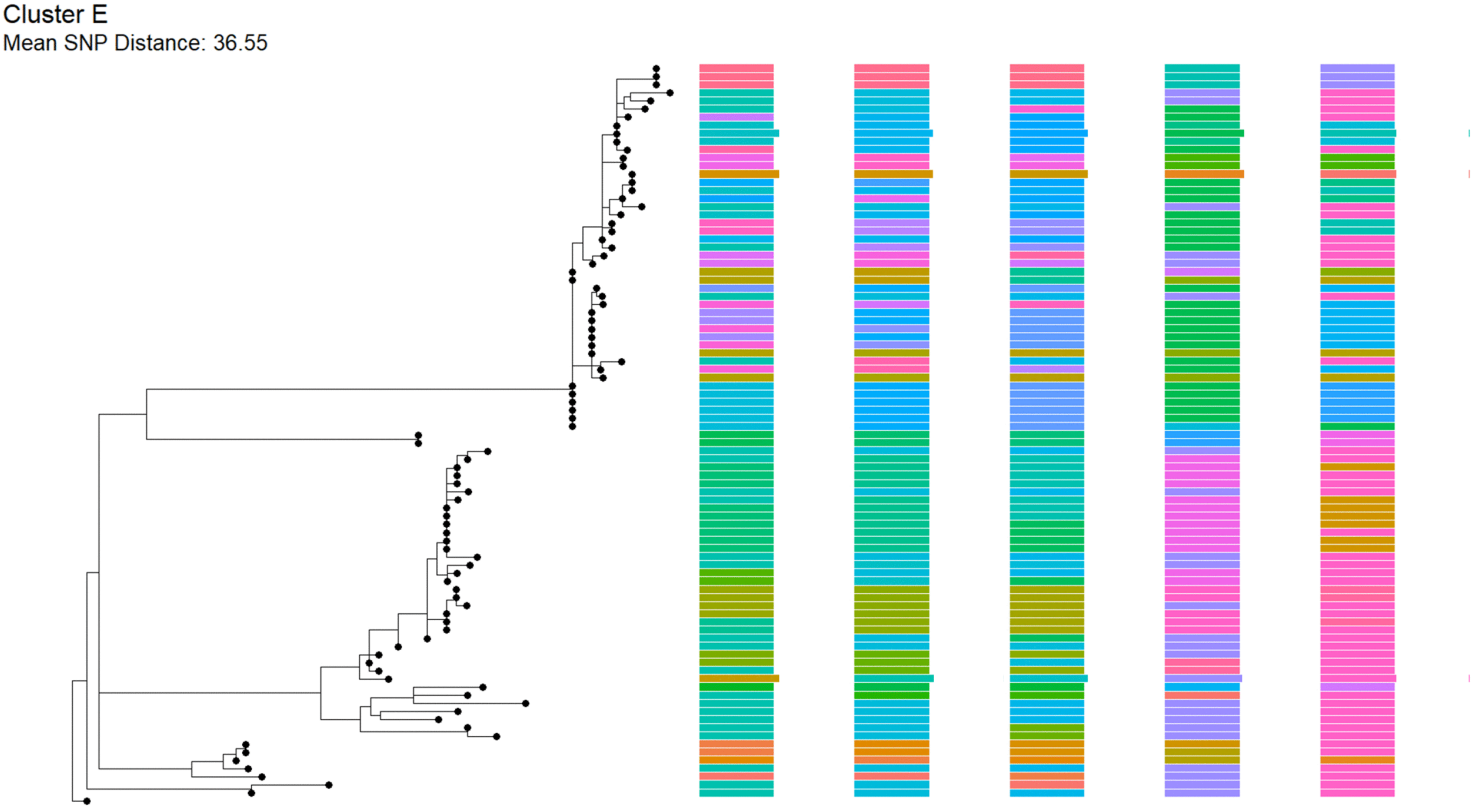
A maximum likelihood phylogenetic tree representing Cluster E, an epidemiologically defined outbreak associated with a correctional facility. Cluster E includes 1,707 cases, of which 93 sequences are represented in this tree. The colors in each column represent cluster membership as identified by each method, including SNP threshold clustering (SNP 1, SNP 2, SNP 3), and logit model clustering using cov2clusters (cov08 and cov09). Gray bars represent sequences that were not part of any cluster according to that method.

Lastly, “Cluster E,” which began in September 2020 in a correctional facility, includes 1,707 cases, though 93 sequences are represented on this tree (Fig 13). This was the largest epidemiologically defined cluster, and the genomic data suggest multiple independent introductions into the prison, similar to what is likely the case in Cluster C. However, because of its size, this cluster in particular underscores the utility of using genomic data in outbreak investigations. Based on the epidemiological data available, these cases may have all seemed equally related, even though most epidemiologists would be able to recognize the improbability of a single cluster of 1,707 cases. With the addition of genomic data, it becomes possible to disentangle the complex transmission patterns, enabling decision makers to potentially intervene in a high risk setting, such as a prison.

## Discussion

This study of 697,393 cases of COVID-19 in Utah found that the odds of reaching a case for whole genome sequencing or manual case investigation differed based on jurisdiction of residence and patient race and ethnicity. Compared to Salt Lake County, the most populous county in the state, patients in most other jurisdictions were more likely to be sequenced, but less likely to be interviewed. Interestingly, some rural jurisdictions with the smallest number of cases overall, like San Juan and Wasatch, had the highest odds of cases being sequenced. The number of sequenced cases from San Juan is likely high in part due to outreach programs specifically targeting that area early in the pandemic. On the other hand, patients identifying as Latino, Native Hawaiian/Pacific Islander, or two or more races were also among the least likely to be sequenced. This suggests a need to prioritize diverse populations for genomic surveillance. Often, genomic surveillance is based on convenience sampling, but during a large outbreak or pandemic, when there are many cases but potentially limited resources, it may be more effective to intentionally prioritize samples that are diverse both geographically and demographically.

There were 8,299 epidemiologically identified clusters between March 2020 and September 2023, with an average of 84 cases per cluster. In contrast, genomic methods, including SNP threshold clustering and logit model clustering, resulted in fewer clusters with fewer cases per cluster on average. The genomic clustering methods produced clusters that were notably different from those identified through traditional epidemiological investigation alone, with adjusted Rand index scores showing poor agreement between epi-identified clusters and genomically-identified clusters. Thus, genomic data may provide additional insight into transmission dynamics that is not captured from traditional epidemiological data alone. This has implications for public health practice; genomic data integrated in real-time surveillance may help identify previously hidden transmission chains by linking seemingly unconnected cases, or prevent the misallocation of resources by refining the inclusion criteria for a disease cluster.

In particular, our data show that genomic data can be a powerful tool for ruling out (rather than ruling in) linkage between cases, which can be helpful to refine the definition of a disease cluster and allow for more targeted, effective public health response. For example, in some instances (such as our Cluster B, as described above), the epidemiologically defined outbreak does seem to reflect the true nature of transmission patterns based on genomic data, but the additional use of genomic data during outbreak investigation could help by removing some cases that are not genomically linked. In other situations (such as Cluster C), the outbreak that was defined epidemiologically actually seems to consist of multiple different outbreaks, or separate disease introductions, which are mistakenly considered to be one large outbreak. And finally, other epidemiologically defined outbreaks do not appear to be outbreaks at all with the addition of genomic data (as in the case of Cluster D). In each of these scenarios, the additional use of genomic data to refine the outbreak definition could influence disease mitigation efforts, particularly in high-risk settings, such as correctional facilities or nursing homes.

Our findings are consistent with other research that has shown that genomic data can improve the detection of transmission clusters, especially in settings like hospitals and nursing homes [13,14,22,23]. For example, Stirrup et al. found that genomic data was able to provide rapid feedback for infection prevention and control (IPC) efforts to control hospital-acquired cases of COVID-19, potentially optimizing IPC in a hospital setting [13]. Similarly, Snell et al. found that genomics was necessary to accurately resolve transmission clusters of COVID-19 in a multi-site healthcare system, especially because unidentified cases of COVID-19 proved to be important vectors of disease spread [14]. On a larger scale, Seemann et al. was able to use genomic data to identify 76 distinct clusters of COVID-19 circulating in Victoria, Australia in the spring of 2020, suggesting that genomics can help guide public health response at a municipal or local government level [5]. Our study builds upon these localized studies by applying these findings at a state level, showing that insights from genomic data are scalable to a broader public health system.

Some studies have shown that genomic data alone offers limited information on transmission dynamics [8,24], and it can be difficult to reconstruct reliable clusters of disease with high transmission rates or low mutation rate [7]. It is true that the mutation rate of SARS-Cov-2 can render it difficult to differentiate true clusters from genomically similar, but epidemiologically unlinked, cases [13]. However, our study supports existing research showing that in combination with epidemiologic data, genomic data can provide added resolution to help clarify transmission dynamics [1,5,13,25].

This study had several limitations. First, cluster validation methods generally showed weak to moderate clustering for each of the genomic methods, which likely reflects the inherent challenges of unsupervised clustering, including subjectivity and a lack of true labels for validation. Without a predefined number of known clusters or a gold standard dataset of true labels for validation, clustering can be messy, and it can be difficult to distinguish true epidemiological transmission chains from background genetic diversity.

Importantly, the low sequencing coverage across epidemiologically identified clusters has significant impacts on both the clustering accuracy and interpretation. Although this analysis included a total of 22,712 sequences, this represents only a fraction of the total cases; fewer than 3% of epidemiologically identified clusters had sequencing data for all cases. This missing data poses significant challenges for clustering algorithms. Our analysis may be prone to artificial fragmentation of true clusters, or false clustering as a result of overestimation of the epidemiological relatedness of sequenced cases. Therefore, our insights into the transmission dynamics must be interpreted with caution. If more comprehensive sequencing data had been available, the clusters resulting from our analysis would likely shift, and our conclusions about transmission dynamics in each cluster may have been different.

Moreover, SARS-CoV-2 was a novel virus, and one that rapidly evolved, which obviously presents challenges to disease surveillance. There was a lot of undetected transmission of the virus throughout the state, even as our disease surveillance efforts adapted to keep up. Even though our genomic clustering approaches, like the SNP distance parameters used, were informed by the SARS-CoV-2 mutation rate and similar analyses in literature [3,7,13,14], this information was largely based on studies done early in the pandemic. While studies have shown that the intra-variant mutation rate remained similar in the three time periods we examined [16,18], it is unclear exactly how small differences in the mutation rate may affect clustering, and the sheer volume of cases during the Omicron period may make it difficult to perform accurate clustering, as there may be genomically similar, but epidemiologically distinct, lineages circulating. We found that the mean silhouette score, a measure of cluster cohesion, decreased from the Alpha period to Omicron for SNP threshold clustering, possibly as a result of differences in the dynamics of the virus at that time. However, the mean silhouette score of the logit model methods actually increased over this time. This is important to consider when applying clustering algorithms to other time periods or different pathogens. To be most useful, clustering methods and parameters should be flexible to adapt to the circumstances of any pathogen or outbreak.

In addition to genomic data challenges, our record of epidemiologically identified clusters is also incomplete. The methods used by Utah epidemiologists to identify and record epidemiological clusters were inconsistent throughout the pandemic. The process changed throughout the course of the pandemic based on workforce availability and the volume of COVID-19 cases. Some epidemiologically-identified clusters may be missing cases or missing entirely from the database. However, this is reflective of the reality of data in state and local public health systems, and a further indication that the added use of genomic data may provide helpful insight. It is expected that our data systems will always be incomplete to some degree, but the additional use of genomic data can help create a clearer picture of disease transmission, despite imperfect data.

Despite these limitations, this study builds on existing evidence that pathogen genomic data can improve the detection and understanding of disease transmission clusters. Unlike many studies on this topic that are hospital- or site-specific and time limited, this is a large study in a state health department setting, offering unique insight into the use of pathogen genomics for public health practice at a state level. These insights may be extremely valuable to health departments or public health practitioners in future pandemics or outbreak scenarios, enabling them to apply lessons learned from COVID-19 to the next pathogen. To that end, we offer the following recommendations as guidelines for the use of genomic data in the future:

**1. Sequence samples that are diverse both geographically and demographically.**

Our data show that certain populations and regions within Utah were less likely to be covered by genomic surveillance. While genomic surveillance efforts often rely on convenience sampling, making efforts to prioritize sequencing cases that will be most representative of the population as a whole will improve the impact of genomic surveillance. This also has relevance to clinical practice as well as public health surveillance; clinical guidelines and treatments like monoclonal antibodies may be more robust if they are developed from a more representative genomic database.

**2. Prioritize sequencing samples thought to be part of a cluster to maximize the information gained from sequencing.**

Genomic data is most powerful in its ability to rule out, rather than confirm, transmission links. If surveillance efforts prioritize sequencing cases that are thought to be part of a cluster, it may be possible to optimize the information gained from sequencing.

**3. Be flexible with genomic clustering methods across pathogens and over time.**

Each pathogen’s unique mutation rate and transmission dynamics will affect the methods used to identify disease clusters or transmission hotspots. Defining the threshold of relatedness as 1 SNP may make sense for one pathogen, but not another, and it may make sense early in a pandemic, but not later.

**4. Use genomic data with any method, either simple or more complex, to help identify and define potential disease clusters.**

SNP distance thresholds represent a computationally simple approach to genomic clustering, while logit models can also account for other variables, such as time or location, potentially offering more refined clustering. No genomic clustering algorithm will be perfect, but any of them will provide more information than epidemiologic data alone, and multiple methods will start to show a general pattern.

## Supporting information

S1 Appendix Accession numbers of sequences included in analysis. (TXT)

S1 File PLOS Human Participants Research Checklist 2025. (PDF)

## References

[1] Gardy JL, Johnston JC, Ho Sui SJ, Cook VJ, Shah L, Brodkin E, et al. Whole-genome sequencing and social-network analysis of a tuberculosis outbreak. N Engl J Med. 2011;364(8):730–9. doi: 10.1056/NEJMoa1003176 21345102

[2] Grubaugh ND, Ladner JT, Lemey P, Pybus OG, Rambaut A, Holmes EC, et al. Tracking virus outbreaks in the twenty-first century. Nat Microbiol. 2019;4(1):10–9. doi: 10.1038/s41564-018-0296-2 30546099 PMC6345516

[3] Suster CJE, Arnott A, Blackwell G, Gall M, Draper J, Martinez E, et al. Guiding the design of SARS-CoV-2 genomic surveillance by estimating the resolution of outbreak detection. Front Public Health. 2022;10:1004201. doi: 10.3389/fpubh.2022.1004201 36276383 PMC9581317

[4] Susvitasari K, Tupper PF, Cancino-Muños I, Lòpez MG, Comas I, Colijn C. Epidemiological cluster identification using multiple data sources: an approach using logistic regression. Microb Genom. 2023;9(3). doi: 10.1099/mgen.0.000929 36867086 PMC10132077

[5] Seemann T, Lane CR, Sherry NL, Duchene S, Gonçalves da Silva A, Caly L, et al. Tracking the COVID-19 pandemic in Australia using genomics. Nat Commun. 2020;11(1):4376. doi: 10.1038/s41467-020-18314-x 32873808 PMC7462846

[6] Sobkowiak B, Kamelian K, Zlosnik JEA, Tyson J, da Silva AG, Hoang LMN, et al. Cov2clusters: genomic clustering of SARS-CoV-2 sequences. BMC Genomics. 2022;23(1):710. doi: 10.1186/s12864-022-08936-4 36258173 PMC9579665

[7] Sobkowiak B, Haghmaram P, Prystajecky N, Zlosnik JEA, Tyson J, Hoang LMN, et al. The utility of SARS-CoV-2 genomic data for informative clustering under different epidemiological scenarios and sampling. Infect Genet Evol. 2023;113:105484. doi: 10.1016/j.meegid.2023.105484 37531976

[8] Gallego-García P, Varela N, Estévez-Gómez N, De Chiara L, Fernández-Silva I, Valverde D, et al. Limited genomic reconstruction of SARS-CoV-2 transmission history within local epidemiological clusters. Virus Evol. 2022;8(1):veac008. doi: 10.1093/ve/veac008 35242361 PMC8889950

[9] McCloskey RM, Poon AFY. A model-based clustering method to detect infectious disease transmission outbreaks from sequence variation. PLoS Comput Biol. 2017;13(11):e1005868. doi: 10.1371/journal.pcbi.1005868 29131825 PMC5703573

[10] Stimson J, Gardy J, Mathema B, Crudu V, Cohen T, Colijn C. Beyond the SNP threshold: identifying outbreak clusters using inferred transmissions. Mol Biol Evol. 2019;36(3):587–603. doi: 10.1093/molbev/msy242 30690464 PMC6389316

[11] Ragonnet-Cronin M, Hodcroft E, Hué S, Fearnhill E, Delpech V, Brown AJL. Automated analysis of phylogenetic clusters. BMC Bioinformatics. 2013;14(1):317. doi: 10.1186/1471-2105-14-317 24191891 PMC4228337

[12] Han AX, Parker E, Maurer-Stroh S, Russell CA. Inferring putative transmission clusters with Phydelity. Virus Evol. 2019;5(2):vez039. doi: 10.1093/ve/vez039 31616568 PMC6785678

[13] Stirrup O, Hughes J, Parker M, Partridge DG, Shepherd JG, Blackstone J. Rapid feedback on hospital onset SARS-CoV-2 infections combining epidemiological and sequencing data. eLife. 2021;10. doi: 10.7554/eLife.65828 PMC8285103 34184637

[14] Snell LB, Fisher CL, Taj U, Stirrup O, Merrick B, Alcolea-Medina A, et al. Combined epidemiological and genomic analysis of nosocomial SARS-CoV-2 infection early in the pandemic and the role of unidentified cases in transmission. Clin Microbiol Infect. 2022;28(1):93–100. doi: 10.1016/j.cmi.2021.07.040 34400345 PMC8361005

[15] Rothman KJ. No adjustments are needed for multiple comparisons. Epidemiology. 1990;1(1):43–6. doi: 10.1097/00001648-199001000-00010 2081237

[16] Ashraf J, Bukhari SARS, Kanji A, Iqbal T, Yameen M, Nisar MI, et al. Substitution spectra of SARS-CoV-2 genome from Pakistan reveals insights into the evolution of variants across the pandemic. Sci Rep. 2023;13(1):20955. doi: 10.1038/s41598-023-48272-5 38017265 PMC10684861

[17] Tay JH, Porter AF, Wirth W, Duchene S. The emergence of SARS-CoV-2 variants of concern is driven by acceleration of the substitution rate. Mol Biol Evol. 2022;39(2):msac013. doi: 10.1093/molbev/msac013 35038741 PMC8807201

[18] Razzaq A, Disoma C, Iqbal S, Nisar A, Hameed M, Qadeer A, et al. Genomic epidemiology and evolutionary dynamics of the Omicron variant of SARS-CoV-2 during the fifth wave of COVID-19 in Pakistan. Front Cell Infect Microbiol. 2024;14:1484637. doi: 10.3389/fcimb.2024.1484637 39502171 PMC11534695

[19] Rousseeuw PJ. Silhouettes: A graphical aid to the interpretation and validation of cluster analysis. J Comput Appl Math. 1987;20:53–65. doi: 10.1016/0377-0427(87)90125-7

[20] Yang Y. Temporal data clustering. Temporal data mining via unsupervised ensemble learning. Elsevier; 2017. pp. 19–34. doi: 10.1016/B978-0-12-811654-8.00003-8

[21] Meilă M. Comparing clusterings—an information based distance. J Multivar Anal. 2007;98(5):873–95. doi: 10.1016/j.jmva.2006.11.013

[22] Meredith LW, Hamilton WL, Warne B, Houldcroft CJ, Hosmillo M, Jahun AS, et al. Rapid implementation of SARS-CoV-2 sequencing to investigate cases of health-care associated COVID-19: a prospective genomic surveillance study. Lancet Infect Dis. 2020;20(11):1263–72. doi: 10.1016/S1473-3099(20)30562-4 32679081 PMC7806511

[23] Roy S, Hartley J, Dunn H, Williams R, Williams CA, Breuer J. Whole-genome sequencing provides data for stratifying infection prevention and control management of nosocomial influenza A. Clin Infect Dis. 2019;69(10):1649–56. doi: 10.1093/cid/ciz020 30993315 PMC6821348

[24] Worby CJ, Lipsitch M, Hanage WP. Within-host bacterial diversity hinders accurate reconstruction of transmission networks from genomic distance data. PLoS Comput Biol. 2014;10(3):e1003549. doi: 10.1371/journal.pcbi.1003549 PMC3967931 24675511

[25] Ypma RJF, Donker T, van Ballegooijen WM, Wallinga J. Finding evidence for local transmission of contagious disease in molecular epidemiological datasets. PLoS One. 2013;8(7):e69875. doi: 10.1371/journal.pone.0069875 23922835 PMC3724731

