# Supplementary material for: Beyond traditional outbreak investigation: Using genomic data for enhanced detection of COVID-19 disease clusters in Utah
Source: PLoS One. 2026 Jul 8;21(7):e0342637. doi: 10.1371/journal.pone.0342637 (PMC13345229; doi:10.1371/journal.pone.0342637)
Supplement: Attachment — Submitted filename: Response to reviewers.docx [file pone.0342637.s004.docx]

Benjamin M. Liu, PhD, D(ABMM), MB(ASCP)

Academic Editor

PLOS One

Dear Dr. Liu,

Thank you for the opportunity to submit a revised draft of my manuscript titled “Beyond traditional outbreak investigation: using genomic data for enhanced detection of COVID-19 disease clusters in Utah” to PLOS One. We are grateful to the reviewers for their insightful comments on the manuscript, and we have made changes to the manuscript (highlighted in red text) based on the feedback.

Here is a point-by-point response to reviewers’ comments:

**Journal Requirements:**

*1. Please ensure that your manuscript meets PLOS ONE's style requirements, including those for file naming.*

We have reviewed PLOS ONE’s style requirements to ensure that we meet all appropriate requirements.

*2. We note that Figure 3 in your submission contain [map/satellite] images which may be copyrighted. All PLOS content is published under the Creative Commons Attribution License (CC BY 4.0), which means that the manuscript, images, and Supporting Information files will be freely available online, and any third party is permitted to access, download, copy, distribute, and use these materials in any way, even commercially, with proper attribution. For these reasons, we cannot publish previously copyrighted maps or satellite images created using proprietary data, such as Google software (Google Maps, Street View, and Earth).*

Thank you for bringing this to our attention. Figure 3 does not contain any proprietary or copyrighted map imagery. This map was generated entirely using an open-source shapefile from the Utah Automated Geographic Reference Center (AGRC), which is explicitly distributed under a Creative Commons Attribution 4.0 International License (CC BY 4.0). We have updated the caption for Figure 3 to explicitly state the source of the data and its CC BY 4.0 status.

*3. Please include captions for your Supporting Information files at the end of your manuscript, and update any in-text citations to match accordingly.*

Thank you. We have included captions for Supporting Information files at the end of the manuscript.

*5. Please review your reference list to ensure that it is complete and correct.*

Thank you for this reminder. We have reviewed the references and they are complete and correct to the best of our knowledge.

**Reviewer Feedback:**

*Reviewer #1: Good provides accurate statistical data and is according to ethics and all data is verified and is valid for publication*.

Thank you for this feedback.

*Reviewer #2: A small elaboration on the clinical relevance or significance of this method and also the implication could add to the knowledge and could also find wider acceptability among all categories of health care workers*.

Thank you for this feedback. We agree that it would be beneficial to elaborate on this point, and have included additional text in the discussion on lines 573-577 and 676-679.

*Reviewer #3: This study successfully combines genetic data with established epidemiological methods to discover COVID-19 clusters in Utah. The strengths are the large dataset (nearly 700,000 cases, with over 22,000 sequences analyzed), the use of multiple clustering approaches (SNP thresholds and logit regression), and the clear demonstration that genomic methods provide higher biological plausibility and more refined cluster definitions than manual case investigation alone. Importantly, the analysis identifies differences in sequencing coverage between jurisdictions and racial/ethnic groups, emphasizing the importance of equitable sampling procedures.*

Thank you for this feedback.

*While the conclusions are strongly supported, the limitations, particularly poor sequencing coverage and diversity in epidemiologic cluster definitions, should be highlighted more, as they affect generalizability.*

We agree with this suggestion. We have included more information on this topic in the paragraph beginning on line 621.

*Reviewer #4: The manuscript is well-structured with vigorous analysis done and data is also well structured. The authors have done a nice work in explaining the trend in COVId-19 and also the statistical methods. Figures are up to the mark.*

Thank you.

*Reviewer #5: 1. The manuscript addresses an important and timely public health question regarding the integration of genomic epidemiology with traditional outbreak investigation for COVID-19 cluster detection.*

*2. A major strength of the study is the large dataset, including approximately 697,000 epidemiologic records and more than 22,000 SARS-CoV-2 genome sequences, which provides strong statistical power and statewide representation.*

*3. The use of multiple genomic clustering approaches, including SNP threshold clustering and the cov2clusters logit regression model, strengthens the methodological framework and allows meaningful comparison between genomic and epidemiologic cluster definitions.*

*4. The inclusion of multiple validation metrics, including silhouette scores, Adjusted Rand Index (ARI), Variation of Information (VI), and monophyly analysis, improves the rigor of the clustering evaluation.*

*5. The findings convincingly demonstrate that genomic clustering methods provide additional resolution compared to traditional epidemiologic investigation and may help refine outbreak definitions.*

*6. The examples involving nursing homes and correctional facilities are particularly informative and effectively demonstrate the practical value of genomic surveillance in outbreak investigations.*

Thank you for this thorough feedback.

*7. The rationale for selecting SNP thresholds (1–3 SNPs) and probability thresholds (0.8 and 0.9) should be explained in greater detail, particularly regarding their applicability across Alpha, Delta, and Omicron periods.*

Thank you for this feedback. We agree that it is important to explain the rationale behind these analytical choices, and we have elaborated on the reasoning to choose these specific thresholds, as well as how these thresholds remain appropriate across all three time periods. This text can be found in lines 147-157 and lines 165-169.

*8. The manuscript should discuss more critically the impact of incomplete sequencing coverage on clustering accuracy and interpretation, especially since only a small proportion of epidemiologic clusters had complete sequencing coverage.*

Thank you, we agree with this suggestion. We have included more information on this topic in the paragraph beginning on line 621.

*9. Additional sensitivity analyses using alternative clustering thresholds or parameters would strengthen the robustness and reproducibility of the conclusions.*

Thank you for this valuable suggestion regarding the robustness of our clustering thresholds. We agree that sensitivity analyses are crucial for demonstrating reproducibility. To ensure the stability of our conclusions, the current manuscript already incorporates sensitivity analyses across five distinct parameter configurations: two probability thresholds (0.8 and 0.9) and three SNP distance thresholds (1, 2, and 3 SNPs). We found that these five levels provided a comprehensive view of the data without overwhelming the reader. We are concerned that introducing additional parameter levels into the main text may compromise the readability and clarity of our manuscript, as well as the figures and tables. However, if the reviewer believes that further thresholds are critical, we would be happy to generate these additional analyses and include them in the Supplementary Material to maintain the streamlined focus of the primary manuscript.

*10. More methodological detail is needed regarding regression assumptions, handling of missing data, assessment of multicollinearity, and whether corrections for multiple testing were considered.*

Thank you for this reminder. We did perform model validations, and we agree that adding these methodological details is necessary. We have added text in the methods section on lines 129-135 to detail the validation process.

*11. Several figures, especially phylogenetic cluster comparisons, would benefit from clearer annotations and more detailed legends to improve accessibility for readers unfamiliar with genomic epidemiology.*

Thank you for this feedback. We have added to our figure legends to improve clarity.

*12. The manuscript is generally well written and understandable; however, portions of the Discussion are repetitive and could be streamlined to improve clarity and readability.*

Thank you for this feedback. We have revised the Discussion section for repetitiveness and tried to improve clarity and readability wherever possible.

*13. Minor grammatical, stylistic, and formatting revisions are recommended to improve overall presentation quality.*

Thank you for this feedback. We have revised the text with this in mind, and we hope that the reviewers and editor will find the revised version substantially improved.

*14. Overall, this is a valuable and technically sound study that contributes meaningfully to the field of genomic epidemiology and public health surveillance. The manuscript would be strengthened further after moderate revision addressing the points above.*

Thank you.

We look forward to hearing from you regarding our submission and to respond to any further questions and comments you may have.

Sincerely,

Mary Jewell
