# Supplementary material for: Beyond traditional outbreak investigation: Using genomic data for enhanced detection of COVID-19 disease clusters in Utah
Source: PLoS One. 2026 Jul 8;21(7):e0342637. doi: 10.1371/journal.pone.0342637 (PMC13345229; doi:10.1371/journal.pone.0342637)
Supplement: Peer review history

# Peer review: Decision Letter 0

**DOI:** 10.1371/journal.pone.0342637.r001

**Authors:** Benjamin Liu

15 May 2026

PONE-D-26-04548

Beyond traditional outbreak investigation: using genomic data for enhanced detection of COVID-19 disease clusters in Utah

PLOS One

Dear Dr. Jewell,

Thank you for submitting your manuscript to PLOS ONE. After careful consideration, we feel that it has merit but does not fully meet PLOS ONE’s publication criteria as it currently stands. Therefore, we invite you to submit a revised version of the manuscript that addresses the points raised during the review process.

Please submit your revised manuscript by Jun 29 2026 11:59PM. If you will need more time than this to complete your revisions, please reply to this message or contact the journal office at plosone@plos.org. When you're ready to submit your revision, log on to https://www.editorialmanager.com/pone/ and select the 'Submissions Needing Revision' folder to locate your manuscript file.

Please include the following items when submitting your revised manuscript:

- A letter that responds to each point raised by the academic editor and reviewer(s). You should upload this letter as a separate file labeled 'Response to Reviewers'.
- A marked-up copy of your manuscript that highlights changes made to the original version. You should upload this as a separate file labeled 'Revised Manuscript with Track Changes'.
- An unmarked version of your revised paper without tracked changes. You should upload this as a separate file labeled 'Manuscript'.

If you would like to make changes to your financial disclosure, please include your updated statement in your cover letter. Guidelines for resubmitting your figure files are available below the reviewer comments at the end of this letter.

If applicable, we recommend that you deposit your laboratory protocols in protocols.io to enhance the reproducibility of your results. Protocols.io assigns your protocol its own identifier (DOI) so that it can be cited independently in the future. For instructions see: https://journals.plos.org/plosone/s/submission-guidelines#loc-laboratory-protocols. Additionally, PLOS ONE offers an option for publishing peer-reviewed Lab Protocol articles, which describe protocols hosted on protocols.io. Read more information on sharing protocols at https://plos.org/protocols?utm_medium=editorial-email&utm_source=authorletters&utm_campaign=protocols.

As the corresponding author, your ORCID iD is verified in the submission system and will appear in the published article. PLOS supports the use of ORCID, and we encourage all coauthors to register for an ORCID iD and use it as well. Please encourage your coauthors to verify their ORCID iD within the submission system before final acceptance, as unverified ORCID iDs will not appear in the published article. *Only*  the individual author can complete the verification step; PLOS staff *cannot*  verify ORCID iDs on behalf of authors.

We look forward to receiving your revised manuscript.

Kind regards,

Benjamin M. Liu, PhD, D(ABMM), MB(ASCP)

Academic Editor

PLOS One

Journal Requirements:

When submitting your revision, we need you to address these additional requirements.

1. Please ensure that your manuscript meets PLOS ONE's style requirements, including those for file naming. The PLOS ONE style templates can be found at

https://journals.plos.org/plosone/s/file?id=wjVg/PLOSOne_formatting_sample_main_body.pdf and

https://journals.plos.org/plosone/s/file?id=ba62/PLOSOne_formatting_sample_title_authors_affiliations.pdf

2. We note that Figure 3 in your submission contain [map/satellite] images which may be copyrighted. All PLOS content is published under the Creative Commons Attribution License (CC BY 4.0), which means that the manuscript, images, and Supporting Information files will be freely available online, and any third party is permitted to access, download, copy, distribute, and use these materials in any way, even commercially, with proper attribution. For these reasons, we cannot publish previously copyrighted maps or satellite images created using proprietary data, such as Google software (Google Maps, Street View, and Earth). For more information, see our copyright guidelines: http://journals.plos.org/plosone/s/licenses-and-copyright.

We require you to either (1) present written permission from the copyright holder to publish these figures specifically under the CC BY 4.0 license, or (2) remove the figures from your submission:

1. You may seek permission from the original copyright holder of Figure 3 to publish the content specifically under the CC BY 4.0 license.

We recommend that you contact the original copyright holder with the Content Permission Form (http://journals.plos.org/plosone/s/file?id=7c09/content-permission-form.pdf) and the following text:

“I request permission for the open-access journal PLOS ONE to publish XXX under the Creative Commons Attribution License (CCAL) CC BY 4.0 (http://creativecommons.org/licenses/by/4.0/). Please be aware that this license allows unrestricted use and distribution, even commercially, by third parties. Please reply and provide explicit written permission to publish XXX under a CC BY license and complete the attached form.”

Please upload the completed Content Permission Form or other proof of granted permissions as an ""Other"" file with your submission.

In the figure caption of the copyrighted figure, please include the following text: “Reprinted from [ref] under a CC BY license, with permission from [name of publisher], original copyright [original copyright year].”

2. If you are unable to obtain permission from the original copyright holder to publish these figures under the CC BY 4.0 license or if the copyright holder’s requirements are incompatible with the CC BY 4.0 license, please either i) remove the figure or ii) supply a replacement figure that complies with the CC BY 4.0 license. Please check copyright information on all replacement figures and update the figure caption with source information. If applicable, please specify in the figure caption text when a figure is similar but not identical to the original image and is therefore for illustrative purposes only.

The following resources for replacing copyrighted map figures may be helpful:

USGS National Map Viewer (public domain): http://viewer.nationalmap.gov/viewer/

The Gateway to Astronaut Photography of Earth (public domain): http://eol.jsc.nasa.gov/sseop/clickmap/

Maps at the CIA (public domain): https://www.cia.gov/library/publications/the-world-factbook/index.html and https://www.cia.gov/library/publications/cia-maps-publications/index.html

NASA Earth Observatory (public domain): http://earthobservatory.nasa.gov/

Landsat: http://landsat.visibleearth.nasa.gov/

USGS EROS (Earth Resources Observatory and Science (EROS) Center) (public domain): http://eros.usgs.gov/#

Natural Earth (public domain): http://www.naturalearthdata.com/

3. Please include captions for your Supporting Information files at the end of your manuscript, and update any in-text citations to match accordingly. Please see our Supporting Information guidelines for more information: http://journals.plos.org/plosone/s/supporting-information.

4. If the reviewer comments include a recommendation to cite specific previously published works, please review and evaluate these publications to determine whether they are relevant and should be cited. There is no requirement to cite these works unless the editor has indicated otherwise.

5. Please review your reference list to ensure that it is complete and correct. If you have cited papers that have been retracted, please include the rationale for doing so in the manuscript text, or remove these references and replace them with relevant current references. Any changes to the reference list should be mentioned in the rebuttal letter that accompanies your revised manuscript. If you need to cite a retracted article, indicate the article’s retracted status in the References list and also include a citation and full reference for the retraction notice.

[Note: HTML markup is below. Please do not edit.]

Reviewers' comments:

Reviewer's Responses to Questions

**Comments to the Author**

1. Is the manuscript technically sound, and do the data support the conclusions?

The manuscript must describe a technically sound piece of scientific research with data that supports the conclusions. Experiments must have been conducted rigorously, with appropriate controls, replication, and sample sizes. The conclusions must be drawn appropriately based on the data presented.

Reviewer #1: Yes

Reviewer #2: Yes

Reviewer #3: Yes

Reviewer #4: Yes

Reviewer #5: Partly

**********

2. Has the statistical analysis been performed appropriately and rigorously?

Reviewer #1: Yes

Reviewer #2: Yes

Reviewer #3: Yes

Reviewer #4: Yes

Reviewer #5: Yes

**********

3. Have the authors made all data underlying the findings in their manuscript fully available?

The PLOS Data policy requires authors to make all data underlying the findings described in their manuscript fully available without restriction, with rare exception (please refer to the Data Availability Statement in the manuscript PDF file). The data should be provided as part of the manuscript or its supporting information, or deposited to a public repository. For example, in addition to summary statistics, the data points behind means, medians and variance measures should be available. If there are restrictions on publicly sharing data—e.g. participant privacy or use of data from a third party—those must be specified.

Reviewer #1: Yes

Reviewer #2: Yes

Reviewer #3: Yes

Reviewer #4: Yes

Reviewer #5: Yes

**********

4. Is the manuscript presented in an intelligible fashion and written in standard English?

PLOS ONE does not copyedit accepted manuscripts, so the language in submitted articles must be clear, correct, and unambiguous. Any typographical or grammatical errors should be corrected at revision, so please note any specific errors here.

Reviewer #1: Yes

Reviewer #2: Yes

Reviewer #3: Yes

Reviewer #4: Yes

Reviewer #5: Yes

**********

5. Review Comments to the Author

Please use the space provided to explain your answers to the questions above. You may also include additional comments for the author, including concerns about dual publication, research ethics, or publication ethics. (Please upload your review as an attachment if it exceeds 20,000 characters)

Reviewer #1: Good provides accurate statistical data and is according to ethics and all data is verified and is valid for publication.This discuss about the people affected by the covid 19 and people vaccinated from covid 19

Reviewer #2: A small elaboration on the clinical relevance or significance of this method and also the implication could add to the knowledge and could also find wider acceptability among all categories of health care workers.

Reviewer #3: This study successfully combines genetic data with established epidemiological methods to discover COVID-19 clusters in Utah. The strengths are the large dataset (nearly 700,000 cases, with over 22,000 sequences analyzed), the use of multiple clustering approaches (SNP thresholds and logit regression), and the clear demonstration that genomic methods provide higher biological plausibility and more refined cluster definitions than manual case investigation alone. Importantly, the analysis identifies differences in sequencing coverage between jurisdictions and racial/ethnic groups, emphasizing the importance of equitable sampling procedures.

While the conclusions are strongly supported, the limitations, particularly poor sequencing coverage and diversity in epidemiologic cluster definitions, should be highlighted more, as they affect generalizability.

Reviewer #4: The manuscript id well-structured with vigorous analysis done and data is also well structured.

The authors have done a nice work in explaining the trend in COVId-19 and also the statistical methods.

Figures are upto the mark.

Reviewer #5: 1. The manuscript addresses an important and timely public health question regarding the integration of genomic epidemiology with traditional outbreak investigation for COVID-19 cluster detection.

2. A major strength of the study is the large dataset, including approximately 697,000 epidemiologic records and more than 22,000 SARS-CoV-2 genome sequences, which provides strong statistical power and statewide representation.

3. The use of multiple genomic clustering approaches, including SNP threshold clustering and the cov2clusters logit regression model, strengthens the methodological framework and allows meaningful comparison between genomic and epidemiologic cluster definitions.

4. The inclusion of multiple validation metrics, including silhouette scores, Adjusted Rand Index (ARI), Variation of Information (VI), and monophyly analysis, improves the rigor of the clustering evaluation.

5. The findings convincingly demonstrate that genomic clustering methods provide additional resolution compared to traditional epidemiologic investigation and may help refine outbreak definitions.

6. The examples involving nursing homes and correctional facilities are particularly informative and effectively demonstrate the practical value of genomic surveillance in outbreak investigations.

7. The rationale for selecting SNP thresholds (1–3 SNPs) and probability thresholds (0.8 and 0.9) should be explained in greater detail, particularly regarding their applicability across Alpha, Delta, and Omicron periods.

8. The manuscript should discuss more critically the impact of incomplete sequencing coverage on clustering accuracy and interpretation, especially since only a small proportion of epidemiologic clusters had complete sequencing coverage.

9. Additional sensitivity analyses using alternative clustering thresholds or parameters would strengthen the robustness and reproducibility of the conclusions.

10. More methodological detail is needed regarding regression assumptions, handling of missing data, assessment of multicollinearity, and whether corrections for multiple testing were considered.

11. Several figures, especially phylogenetic cluster comparisons, would benefit from clearer annotations and more detailed legends to improve accessibility for readers unfamiliar with genomic epidemiology.

12. The manuscript is generally well written and understandable; however, portions of the Discussion are repetitive and could be streamlined to improve clarity and readability.

13. Minor grammatical, stylistic, and formatting revisions are recommended to improve overall presentation quality.

14. Overall, this is a valuable and technically sound study that contributes meaningfully to the field of genomic epidemiology and public health surveillance. The manuscript would be strengthened further after moderate revision addressing the points above.

**********

6. PLOS authors have the option to publish the peer review history of their article (what does this mean?). If published, this will include your full peer review and any attached files.

If you choose “no”, your identity will remain anonymous but your review may still be made public.

**Do you want your identity to be public for this peer review?**  For information about this choice, including consent withdrawal, please see our Privacy Policy.

Reviewer #1: **Yes:**  Kunal Joon

Reviewer #2: **Yes:**  Dr. Bineeta Kashyap

Reviewer #3: **Yes:**  Venkataramana Kandi

Reviewer #4: **Yes:**  Dr. Asiya Khan

Reviewer #5: No

**********

[NOTE: If reviewer comments were submitted as an attachment file, they will be attached to this email and accessible via the submission site. Please log into your account, locate the manuscript record, and check for the action link "View Attachments". If this link does not appear, there are no attachment files.]

To ensure your figures meet our technical requirements, please review our figure guidelines: https://journals.plos.org/plosone/s/figures

You may also use PLOS’s free figure tool, NAAS, to help you prepare publication quality figures: https://journals.plos.org/plosone/s/figures#loc-tools-for-figure-preparation.

NAAS will assess whether your figures meet our technical requirements by comparing each figure against our figure specifications.

# Author comment: Author response to Decision Letter 1

**DOI:** 10.1371/journal.pone.0342637.r002

10 Jun 2026

Benjamin M. Liu, PhD, D(ABMM), MB(ASCP)

Academic Editor

PLOS One

Dear Dr. Liu,

Thank you for the opportunity to submit a revised draft of my manuscript titled “Beyond traditional outbreak investigation: using genomic data for enhanced detection of COVID-19 disease clusters in Utah” to PLOS One. We are grateful to the reviewers for their insightful comments on the manuscript, and we have made changes to the manuscript (highlighted in red text) based on the feedback.

Here is a point-by-point response to reviewers’ comments:

Journal Requirements:

1. Please ensure that your manuscript meets PLOS ONE's style requirements, including those for file naming.

We have reviewed PLOS ONE’s style requirements to ensure that we meet all appropriate requirements.

2. We note that Figure 3 in your submission contain [map/satellite] images which may be copyrighted. All PLOS content is published under the Creative Commons Attribution License (CC BY 4.0), which means that the manuscript, images, and Supporting Information files will be freely available online, and any third party is permitted to access, download, copy, distribute, and use these materials in any way, even commercially, with proper attribution. For these reasons, we cannot publish previously copyrighted maps or satellite images created using proprietary data, such as Google software (Google Maps, Street View, and Earth).

Thank you for bringing this to our attention. Figure 3 does not contain any proprietary or copyrighted map imagery. This map was generated entirely using an open-source shapefile from the Utah Automated Geographic Reference Center (AGRC), which is explicitly distributed under a Creative Commons Attribution 4.0 International License (CC BY 4.0). We have updated the caption for Figure 3 to explicitly state the source of the data and its CC BY 4.0 status.

3. Please include captions for your Supporting Information files at the end of your manuscript, and update any in-text citations to match accordingly.

Thank you. We have included captions for Supporting Information files at the end of the manuscript.

5. Please review your reference list to ensure that it is complete and correct.

Thank you for this reminder. We have reviewed the references and they are complete and correct to the best of our knowledge.

Reviewer Feedback:

Reviewer #1: Good provides accurate statistical data and is according to ethics and all data is verified and is valid for publication.

Thank you for this feedback.

Reviewer #2: A small elaboration on the clinical relevance or significance of this method and also the implication could add to the knowledge and could also find wider acceptability among all categories of health care workers.

Thank you for this feedback. We agree that it would be beneficial to elaborate on this point, and have included additional text in the discussion on lines 573-577 and 676-679.

Reviewer #3: This study successfully combines genetic data with established epidemiological methods to discover COVID-19 clusters in Utah. The strengths are the large dataset (nearly 700,000 cases, with over 22,000 sequences analyzed), the use of multiple clustering approaches (SNP thresholds and logit regression), and the clear demonstration that genomic methods provide higher biological plausibility and more refined cluster definitions than manual case investigation alone. Importantly, the analysis identifies differences in sequencing coverage between jurisdictions and racial/ethnic groups, emphasizing the importance of equitable sampling procedures.

Thank you for this feedback.

While the conclusions are strongly supported, the limitations, particularly poor sequencing coverage and diversity in epidemiologic cluster definitions, should be highlighted more, as they affect generalizability.

We agree with this suggestion. We have included more information on this topic in the paragraph beginning on line 621.

Reviewer #4: The manuscript is well-structured with vigorous analysis done and data is also well structured. The authors have done a nice work in explaining the trend in COVId-19 and also the statistical methods. Figures are up to the mark.

Thank you.

Reviewer #5: 1. The manuscript addresses an important and timely public health question regarding the integration of genomic epidemiology with traditional outbreak investigation for COVID-19 cluster detection.

2. A major strength of the study is the large dataset, including approximately 697,000 epidemiologic records and more than 22,000 SARS-CoV-2 genome sequences, which provides strong statistical power and statewide representation.

3. The use of multiple genomic clustering approaches, including SNP threshold clustering and the cov2clusters logit regression model, strengthens the methodological framework and allows meaningful comparison between genomic and epidemiologic cluster definitions.

4. The inclusion of multiple validation metrics, including silhouette scores, Adjusted Rand Index (ARI), Variation of Information (VI), and monophyly analysis, improves the rigor of the clustering evaluation.

5. The findings convincingly demonstrate that genomic clustering methods provide additional resolution compared to traditional epidemiologic investigation and may help refine outbreak definitions.

6. The examples involving nursing homes and correctional facilities are particularly informative and effectively demonstrate the practical value of genomic surveillance in outbreak investigations.

Thank you for this thorough feedback.

7. The rationale for selecting SNP thresholds (1–3 SNPs) and probability thresholds (0.8 and 0.9) should be explained in greater detail, particularly regarding their applicability across Alpha, Delta, and Omicron periods.

Thank you for this feedback. We agree that it is important to explain the rationale behind these analytical choices, and we have elaborated on the reasoning to choose these specific thresholds, as well as how these thresholds remain appropriate across all three time periods. This text can be found in lines 147-157 and lines 165-169.

8. The manuscript should discuss more critically the impact of incomplete sequencing coverage on clustering accuracy and interpretation, especially since only a small proportion of epidemiologic clusters had complete sequencing coverage.

Thank you, we agree with this suggestion. We have included more information on this topic in the paragraph beginning on line 621.

9. Additional sensitivity analyses using alternative clustering thresholds or parameters would strengthen the robustness and reproducibility of the conclusions.

Thank you for this valuable suggestion regarding the robustness of our clustering thresholds. We agree that sensitivity analyses are crucial for demonstrating reproducibility. To ensure the stability of our conclusions, the current manuscript already incorporates sensitivity analyses across five distinct parameter configurations: two probability thresholds (0.8 and 0.9) and three SNP distance thresholds (1, 2, and 3 SNPs). We found that these five levels provided a comprehensive view of the data without overwhelming the reader. We are concerned that introducing additional parameter levels into the main text may compromise the readability and clarity of our manuscript, as well as the figures and tables. However, if the reviewer believes that further thresholds are critical, we would be happy to generate these additional analyses and include them in the Supplementary Material to maintain the streamlined focus of the primary manuscript.

10. More methodological detail is needed regarding regression assumptions, handling of missing data, assessment of multicollinearity, and whether corrections for multiple testing were considered.

Thank you for this reminder. We did perform model validations, and we agree that adding these methodological details is necessary. We have added text in the methods section on lines 129-135 to detail the validation process.

11. Several figures, especially phylogenetic cluster comparisons, would benefit from clearer annotations and more detailed legends to improve accessibility for readers unfamiliar with genomic epidemiology.

Thank you for this feedback. We have added to our figure legends to improve clarity.

12. The manuscript is generally well written and understandable; however, portions of the Discussion are repetitive and could be streamlined to improve clarity and readability.

Thank you for this feedback. We have revised the Discussion section for repetitiveness and tried to improve clarity and readability wherever possible.

13. Minor grammatical, stylistic, and formatting revisions are recommended to improve overall presentation quality.

Thank you for this feedback. We have revised the text with this in mind, and we hope that the reviewers and editor will find the revised version substantially improved.

14. Overall, this is a valuable and technically sound study that contributes meaningfully to the field of genomic epidemiology and public health surveillance. The manuscript would be strengthened further after moderate revision addressing the points above.

Thank you.

We look forward to hearing from you regarding our submission and to respond to any further questions and comments you may have.

Sincerely,

Mary Jewell

Attachment Submitted filename: Response to reviewers.docx

# Editor report: Decision Letter 1

**DOI:** 10.1371/journal.pone.0342637.r003

**Authors:** Benjamin Liu, Benjamin Liu

21 Jun 2026

Beyond traditional outbreak investigation: using genomic data for enhanced detection of COVID-19 disease clusters in Utah

PONE-D-26-04548R1

Dear Dr. Jewell,

We’re pleased to inform you that your manuscript has been judged scientifically suitable for publication and will be formally accepted for publication once it meets all outstanding technical requirements.

Within one week, you’ll receive an e-mail detailing the required amendments. When these have been addressed, you’ll receive a formal acceptance letter and your manuscript will be scheduled for publication.

An invoice will be generated when your article is formally accepted. Please note, if your institution has a publishing partnership with PLOS and your article meets the relevant criteria, all or part of your publication costs will be covered. Please make sure your user information is up-to-date by logging into Editorial Manager at Editorial Manager® and clicking the ‘Update My Information' link at the top of the page. For questions related to billing, please contact billing support.

If your institution or institutions have a press office, please notify them about your upcoming paper to help maximize its impact. If they’ll be preparing press materials, please inform our press team as soon as possible -- no later than 48 hours after receiving the formal acceptance. Your manuscript will remain under strict press embargo until 2 pm Eastern Time on the date of publication. For more information, please contact onepress@plos.org.

Kind regards,

Benjamin M. Liu, PhD, D(ABMM), MB(ASCP)

Academic Editor

PLOS One

Additional Editor Comments (optional):

Reviewers' comments:

# Editor report: Acceptance letter

**DOI:** 10.1371/journal.pone.0342637.r004

**Authors:** Benjamin Liu, Benjamin Liu

PONE-D-26-04548R1

PLOS One

Dear Dr. Jewell,

I'm pleased to inform you that your manuscript has been deemed suitable for publication in PLOS One. Congratulations! Your manuscript is now being handed over to our production team.

At this stage, our production department will prepare your paper for publication. This includes ensuring the following:

* All references, tables, and figures are properly cited

* All relevant supporting information is included in the manuscript submission,

* There are no issues that prevent the paper from being properly typeset

You will receive further instructions from the production team, including instructions on how to review your proof when it is ready. Please keep in mind that we are working through a large volume of accepted articles, so please give us a few days to review your paper and let you know the next and final steps.

Lastly, if your institution or institutions have a press office, please let them know about your upcoming paper now to help maximize its impact. If they'll be preparing press materials, please inform our press team within the next 48 hours. Your manuscript will remain under strict press embargo until 2 pm Eastern Time on the date of publication. For more information, please contact onepress@plos.org.

You will receive an invoice from PLOS for your publication fee after your manuscript has reached the completed accept phase. If you receive an email requesting payment before acceptance or for any other service, this may be a phishing scheme. Learn how to identify phishing emails and protect your accounts at https://explore.plos.org/phishing.

If we can help with anything else, please email us at customercare@plos.org.

Thank you for submitting your work to PLOS ONE and supporting open access.

Kind regards,

PLOS ONE Editorial Office Staff

on behalf of

Dr. Benjamin M. Liu

Academic Editor

PLOS One
